# Semantic integration of gene expression analysis tools and data sources using software connectors

**DOI:** 10.1186/1471-2164-14-S6-S2

**Published:** 2013-10-25

**Authors:** Flávia A Miyazaki, Gabriela DA Guardia, Ricardo ZN Vêncio, Cléver RG de Farias

**Affiliations:** 1 Department of Computer Science and Mathematics (DCM/FFCLRP), University of São Paulo (USP) Av. Bandeirantes, 3900 - Monte Alegre - Ribeirão Preto - SP - 14040-901 - Brazil

## Abstract

**Background:**

The study and analysis of gene expression measurements is the primary focus of functional genomics. Once expression data is available, biologists are faced with the task of extracting (new) knowledge associated to the underlying biological phenomenon. Most often, in order to perform this task, biologists execute a number of analysis activities on the available gene expression dataset rather than a single analysis activity. The integration of heteregeneous tools and data sources to create an integrated analysis environment represents a challenging and error-prone task. Semantic integration enables the assignment of unambiguous meanings to data shared among different applications in an integrated environment, allowing the exchange of data in a semantically consistent and meaningful way. This work aims at developing an ontology-based methodology for the semantic integration of gene expression analysis tools and data sources. The proposed methodology relies on software connectors to support not only the access to heterogeneous data sources but also the definition of transformation rules on exchanged data.

**Results:**

We have studied the different challenges involved in the integration of computer systems and the role software connectors play in this task. We have also studied a number of gene expression technologies, analysis tools and related ontologies in order to devise basic integration scenarios and propose a reference ontology for the gene expression domain. Then, we have defined a number of activities and associated guidelines to prescribe how the development of connectors should be carried out. Finally, we have applied the proposed methodology in the construction of three different integration scenarios involving the use of different tools for the analysis of different types of gene expression data.

**Conclusions:**

The proposed methodology facilitates the development of connectors capable of semantically integrating different gene expression analysis tools and data sources. The methodology can be used in the development of connectors supporting both simple and nontrivial processing requirements, thus assuring accurate data exchange and information interpretation from exchanged data.

## Background

Gene expression is the biological process in which the information stored in a gene is converted into protein or RNA. Nowadays, high-throughput expression measurements of entire transcriptomes can be easily obtained through different techniques. These techniques differ from each other on the technology and/or approach used to obtain a measurement. Nonetheless, they can roughly be classified as: hibridization (e.g., Microarray [1] and Tiling Array [2]), sequencing (e.g., ESTs [3], SAGE [4], MPSS [5] and RNA-Seq [6]) and other non high-throughput approaches (e.g., Northern Blot [7], qPCR [8] and cDNA Macroarray [9]).

The study and analysis of these measurements is the primary focus of functional genomics [10]. Overall, the objectives of functional genomics include the definition of the functional roles of different genes and associated processes, the study of the interactions between gene and gene products, the study of gene expression variations in different cell types and under different conditions, among others [11]. Once expression data is available, biologists are faced with the task of extracting (new) knowledge associated to the underlying biological phenomenon. Most often, in order to carry out this task, biologists perform a sequence of analysis activities on the available gene expression dataset rather than a single analysis activity. Analysis activities include data normalization, identification of differentially expressed genes, pathway analysis, cluster analysis and classification, functional annotation and modelling gene regulatory networks.

Occasionally, an integrated analysis environment, also commonly refered as an integrated analysis pipeline (e.g., [12-17]), can be used to support the integrated execution of different analysis activities. However, in most cases biologists have to use different, isolated analysis tools. In those cases, biologists must frequently implement themselves, without proper guidelines, the necessary code to integrate those tools, which can hinder effective research [18].

The integration of complete systems or part of systems to build a new system is a challenging task, even when these systems were developed using the same programming language and the same execution platform. System integration can be achieved at syntactical and semantical levels. On the one hand, the syntactic integration aims at agreeing on a common syntax for representing the data exchanged between different parts of the system. On the other hand, the semantic integration aims at agreeing on common meanings for the data exchanged, under possibly different syntactical formats, between different parts of the system. Semantic integration is usually accomplished using a neutral (reference) ontology to enable the translation first from the source system format into the neutral format and then, from there, into the target system format [19]. Thus, semantic integration enables the assignment of unambiguous meanings to data intended to be shared among different systems in an integrated environment, allowing the exchange of data in a semantically consistent and meaningful way.

In the gene expression domain, the emerging of high-throughput experimental processes has lead to development of different (standard) formats for the representation and exchange of gene expression data, such as MAGE-ML [20], MAGE-TAB [21], SOFT [22], MINiML [23], SAM [24] and FuGE-ML [25,26]. However, the availability of a standard data representation format is not enough to guarantee semantic integration of heterogenous tools because not all tools comply with these formats, different types of data can be represented using a single format and, perhaps most importantly, analysis activities can be carried out without proper reasoning about the meaning of exchanged data, among others. For example, we can store and exchange both one-color and two-color microarray data according to the MAGE-TAB data format. However, any analysis activity carried out on these data should take into account its meaning in order to obtain biologically significant results. Thus, we should not normalize one-color microarray data in the same way as we normalize two-color microarray data.

Ontologies have been used to facilitate the integration of computer systems and information in the biomedical domain. Particularly, we can identify two general ontology-based approaches for the integration of bioinformatics systems and databases [27]. In the first approach, ontologies have been used as source for the design of a common or reference (database) model shared by a number of related tools or databases (e.g., [20,28-32]). In the second approach, ontologies have been used as basis for the development of mediators, i.e., software entities that encompasses a global knowledge (global database schema) and, at the same time, are able to provide mappings to the specific (local) schemas to be integrated (e.g., [33-36]).

In the gene expression domain, different and often complementary activities are usually carried out using different tools during the analysis process. Additionally, new biological and experimental developments frequently lead to the modification of existing data models and the development of new algorithms and analysis tools. Thus, both approaches pose a number of limitations for the integration of gene expression analysis tools. The first approach lacks flexibility, since the integration of a new tool requires the adaptation of this tool data model to the reference model and/or the modification (extension) of the reference model. The second approach lacks generality, since its primarily focus lies on the translation of queries between a mediator and local schemas. Finally, none of the approaches supports dynamic processing (transformation) of the exchanged data, which is often necessary to enable the proper usage of data by a target tool.

This work aims at developing an ontology-based methodology for the semantic integration of gene expression analysis tools to support not only the access to heterogeneous data sources but also the definition of transformation rules on exchanged data. We have used software connectors as basis for our integrative solution. Software connectors represent architectural elements used to model interactions among either computation or data components of a system. We have studied the different challenges involved in the integration of computer systems and the role connectors play in this task. We have also studied a number of gene expression technologies, analysis tools and related ontologies in order to devise basic integration scenarios and propose a gene expression domain ontology. Finally, we have proposed a number of activities and associated guidelines to develop connectors. This methodology was then applied in the construction of a number of integration scenarios involving different gene expression data and/or tools. The proposed methodology allows the development of connectors capable of integrating different gene expression analysis tools and/or related data at a semantic level, thus assuring accurate data exchange and information interpretation from the exchanged data. Additionally, our methodology can be used in the development of connectors supporting both simple and nontrivial processing requirements.

## Methods

The following steps were carried out in the development of our ontology-based methodology: 1) study of software architecture and integration of computer systems; 2) study of gene expression technologies, analysis tools and related ontologies; 3) definition of a reference ontology for the gene expression domain; 4) definition of activities and associated guidelines for connector development, and; 5) application of the proposed methodology in the construction of different integration scenarios.

### Software architecture

Software architecture emmerged as a sub-discipline of software engeneering in the early 90's. This discipline is focused, among others, on the architecture description of complex systems and on the use of this description as basis for system design, development, reuse and management in general [37]. The architecture of a software system represents the fundamental properties of this system in relation to its enviroment, embodied in its elements, relationships, and in the principles of its design and evolution [38] (cf. [37,39-43]). An architecture is represented by means of an architecture description. Architectural Description Languages (ADLs) can be used to create an architecture description through the development of one or more architecture models (see [44] for an overview and comparison of ADLs).

Despite existing diferences among ADLs, there seems to be general agreement with respect to the elements needed to describe structural aspects of an architecture, viz., components, connectors and architectural configurations [44]. A component represents a unit of computation or data store (state) in a system. A component can be as simple as a single procedure or method or as complex as an entire application. A connector represents an architectural element used to model interactions among components and rules governing those interactions. Connectors also specify any auxiliary mechanism required to perform the interactions [45]. A connector can be as simple as a global variable (shared memory) or procedure call or as complex as a P2P-based data distribution connector [43]. An architectural configuration represents a set of associations between components and connectors pertaining to a system's architecture. An architectural configuration can be represented simply as a graph whose nodes represent components and connectors and whose edges represent their associations Ibid.

Connectors are built based on basic primitives for transferring data and/or control. Connectors can be classified according to the set of provided services. Four basic services can be identified [43,45]: *communication*, which supports transmission of data among components; *coordination*, which supports transfer of control among components; *conversion*, which transforms the interaction provided by one component to the interaction required by another; and *facilitation*, which mediates and streamlines component interaction in order to optimize interactions and reduce interdependencies. Simple connectors, which are implemented directly in programming languages, provide a single service, while composite connectors provide multiple services, often exibiting an internal architecture with computation and data storage capabilities.

Most connectors provide multiple services. For example, a procedure call connector provides both communication and coordenation services, while a P2P-based data distribution connector provides all four types of services. In the context of this work, the developed connectors provide a combination of communication, coordination and conversion services in general.

### Gene expression analysis

In order to apply the proposed methodology we have devised three integration scenarios for the analysis of different types of gene expression data. Each scenario involves the integration of different tools and/or data sources. The following tools were considered: R *Environment Graphical User Interface *(*RGUI*) [46,47], which was used for microarray data normalization and identification of differentially expressed genes; KEGG Mapper - Search&Color Pathway [48], which was used for searching KEGG pathway maps using differentially expressed genes and then coloring regulation accordingly; *DataMatrixViewer *(*DMV*) [49], which was used for RNA-Seq data selection and displaying; *TIGR MultiExperiment Viewer *(*TMeV*) [50], which was used for RNA-Seq data clustering; and *DAVID Bioinformatics Resources *(*DAVID*) [51], which was used for gene functional classification.

Each proposed integration scenario involves the analysis of different source gene expression data in order to reproduce (part of) different studies already documented in the literature. In the first scenario, two-color *Plasmodium vivax *microarray data [52], available from NCBI Gene Expression Omnibus (GEO) under the accession number GSE11075, were initially normalized and then analysed for differentially expressed genes using RGUI. In the sequel, KEGG *Plasmodium vivax *pathways were analysed using KEGG Mapper - Search&Color Pathway. This tool was used to identify which parts of pathways are associated with the list of differentially expressed genes provided by RGUI, highlighting up and down regulation according to a user-defined color scheme. In the second integration scenario, *Sulfolobus solfataricus *cDNA sequencing data, obtained from a RNA-Seq platform [53], available from GEO under the accession number GSE18630 and aligned with Bowtie [54], were initially filtered using DMV and then clusterized using TMev. Finally, in the third integration scenario, one-color microarray data taken from normal and cancer prostate cells [55], also available from GEO under the accession number GSE17906, were analysed to find differentially expressed genes using a RGUI implementation of HTself [56], a self-self based statistical method for low replication microarray data. The obtained data were then loaded into DAVID for functional analysis.

Automatic interaction with RGUI from a third-party application was provided by the RServe API [57] (http://rforge.net/org/doc/org/rosuda/REngine/Rserve/package-summary.html). This API allows the establishment of a (remote) communication connection (using TCP) between the R system and a Java application. This connection was then used to send R commands to be processed by the R system and, after their execution, to receive the corresponding answers.

### Biomedical ontologies

Many different ontologies have been proposed in the biomedical domain. The Open Biological and Biomedical Ontologies (OBO) Foundry is a consortium that provides a repository of life-science ontologies [58]. These ontologies have been developed according to a set of shared principles, including openness, orthogonality and collaborative development. The OBO Foundry ontologies include the Gene Ontology (GO) [59], the Chemical Entities of Biological Interest Ontology (ChEBI) [60], the Phenotypic Quality Ontology (PATO) [61] and the PRotein Ontology (PRO) [62], among others. Additionally, the Foundry also includes a number of candidate ontologies and other ontologies of interest in the life-science domain, such as the Sequence Ontology (SO) [63], the Common Anatomy Reference Ontology (CARO) [64] and the Ontology for Biomedical Investigations (OBI) [65].

The proposed methodology uses a reference ontology for the gene expression domain called Gene Expression Ontology (GEXPO). Two ontologies were considered in the development of our reference ontology: Gene Ontology (GO) [59] and Sequence Ontology (SO) [63]. GO provides a set of terms and relations used for standardization of genes and their products in eukaryotic organisms using three independent ontologies: *Cellular Component*, which describes cellular structures in which genes can be expressed; *Molecular Function*, which describes activities that occur at the molecular level; and *Biological Process*, which describes collections of processes (series of events or molecular functions) related to the functioning of integrated living units. SO provides a set of terms and relations used to describe features and attributes of biological sequences. The development of such controlled terminology aims at facilitating the exchange, analysis and management of genomic data, particularly genomic databases and flat file data exchange formats.

Ontologies can be represented using different languages, such as the Unified Modeling Language (UML) [66,67], the Web Ontology Language (OWL) [68] and the OBO Flat File Format [69]. UML is a standard graphical language widely used in the specification, documentation and visualization of computer artifacts and ontologies. OWL is an ontology definition language originally conceived for the semantic web. OWL specifications are serialized using a machine-readable RDF/XML-based format. The OBO Flat File Format or simply OBO format is also a machine-readable, text-based ontology representation language. The OBO format provides a subset of the concepts in OWL, with a number of extensions.

Our gene expression reference ontology has been represented using OWL. In order to facilitate the visualization of the proposed ontology and the understanding of the integration scenarios, we have also created UML representations of parts of our reference ontology.

## Results

### Basic integration scenarios

In order to develop our methodology, we initially had to identify a set of basic scenarios in which integration could take place.

**Figure 1:**
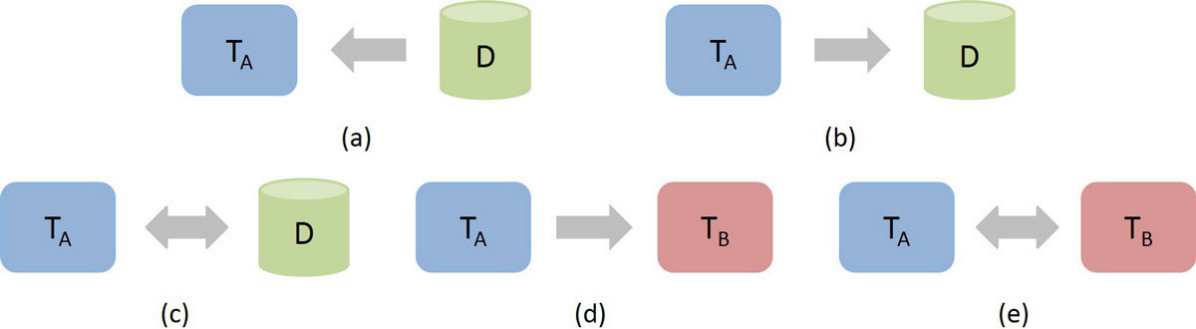
**Basic integration scenarios**. A cylinder represents a data source, while a rectangle with rounded corners represents a tool. An one-way arrow represents a directed flow of data and/or control, while a two-way arrow represents a bidirectional flow of data and/or control.

A tool *T_A _*can be integrated to another tool *T_B _*or to a data source *D *in different ways, considering both the transfer of data and/or control. In this sense, five basic integration scenarios can be identified (see Figure 1): (a) data stored in D are transferred to T*_A_*; (b) data produced by T*_A _*are transferred to D; (c) data stored in D are transferred to T*_A _*and later data produced by T*_A _*are transferred back to D; (d) data and/or control from T*_A _*are transferred to T*_B_*; and (e) data and/or control from T*_A _*are transferred to T*_B _*and later data and/or control from T*_B _*are transferred back to T*_A_*.

Integration scenarios *c *and *e *can be considered composite integration scenarios since they can be structured as a composition of the other (simpler) scenarios (*a, b *and *d*). Integration scenario *c *can be considered a combination of scenarios *a *and *b*, while integration scenario *e *can be considered a combination of two scenarios *d*. In both cases, there is a bidirectional flow of data between a data source and a tool or a bidirectional flow of data and/or control between two tools. In this sense, the guidelines provided in this work take into account only unidirectional scenarios (*a, b *and *d*), but can be easily extended to cover bidirectional scenarios.

### Gene Expression Ontology (GEXPO)

Different ontologies have been proposed in the biomedical domain. These ontologies usually focus on specific aspects of this domain, such as biological processes, cellular components, molecular functions, biological sequences, etc. However, there is no ontology whose primary focus is the gene expression domain, although many concepts of this domain are present in different ontologies. So, after identifying the basic integration scenarios we developed a reference ontology, called Gene Expression Ontology (GEXPO), to be used as basis for semantic integration.

**Figure 2:**
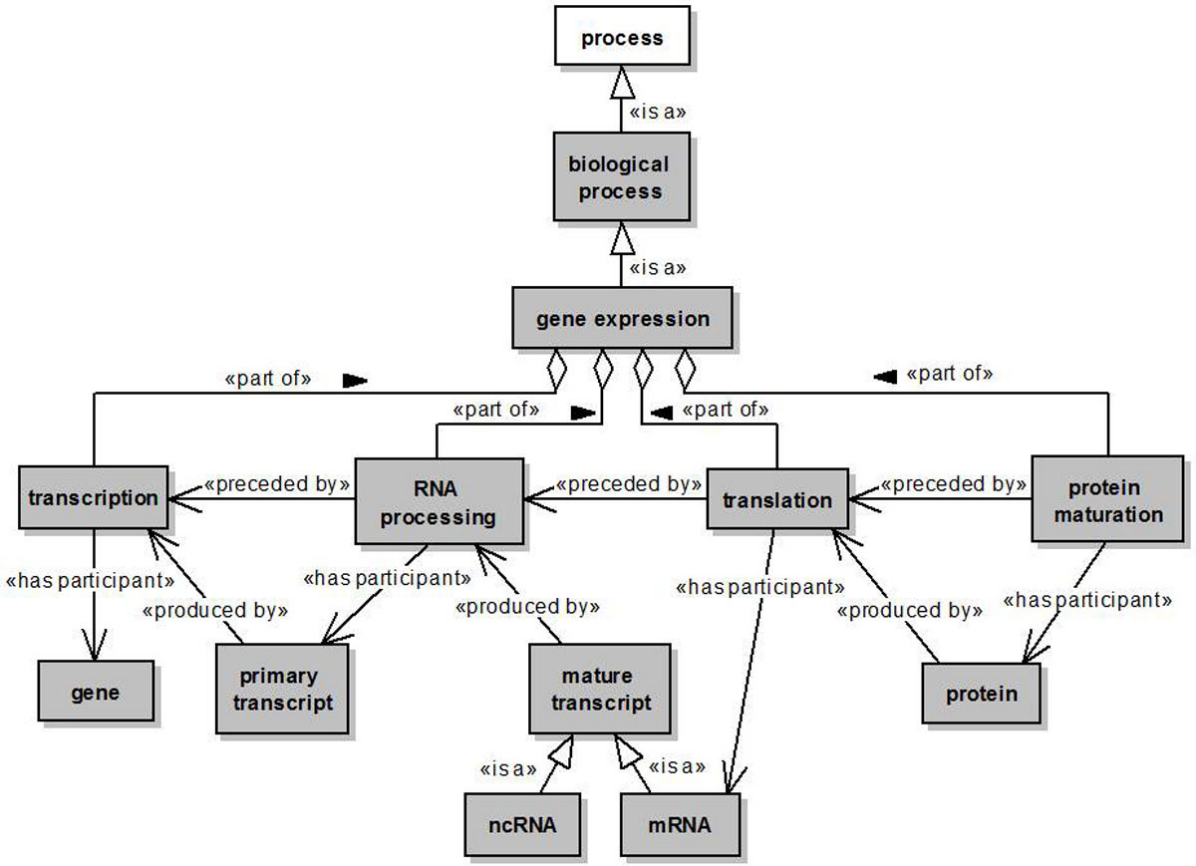
**Gene expression process**. A named rectangle represents a concept. A white rectangle represents a concept introduced in our ontology, while a gray rectangle represents a concept reused from another ontology. A solid line with a stick arrowhead at its end represents a relationship between two concepts. A solid line with a hollow diamond at its end represents a *part of *relationship. Finally, a solid line with a hollow triangle as an arrowhead represents an *is a *relationship.

**Figure 3:**
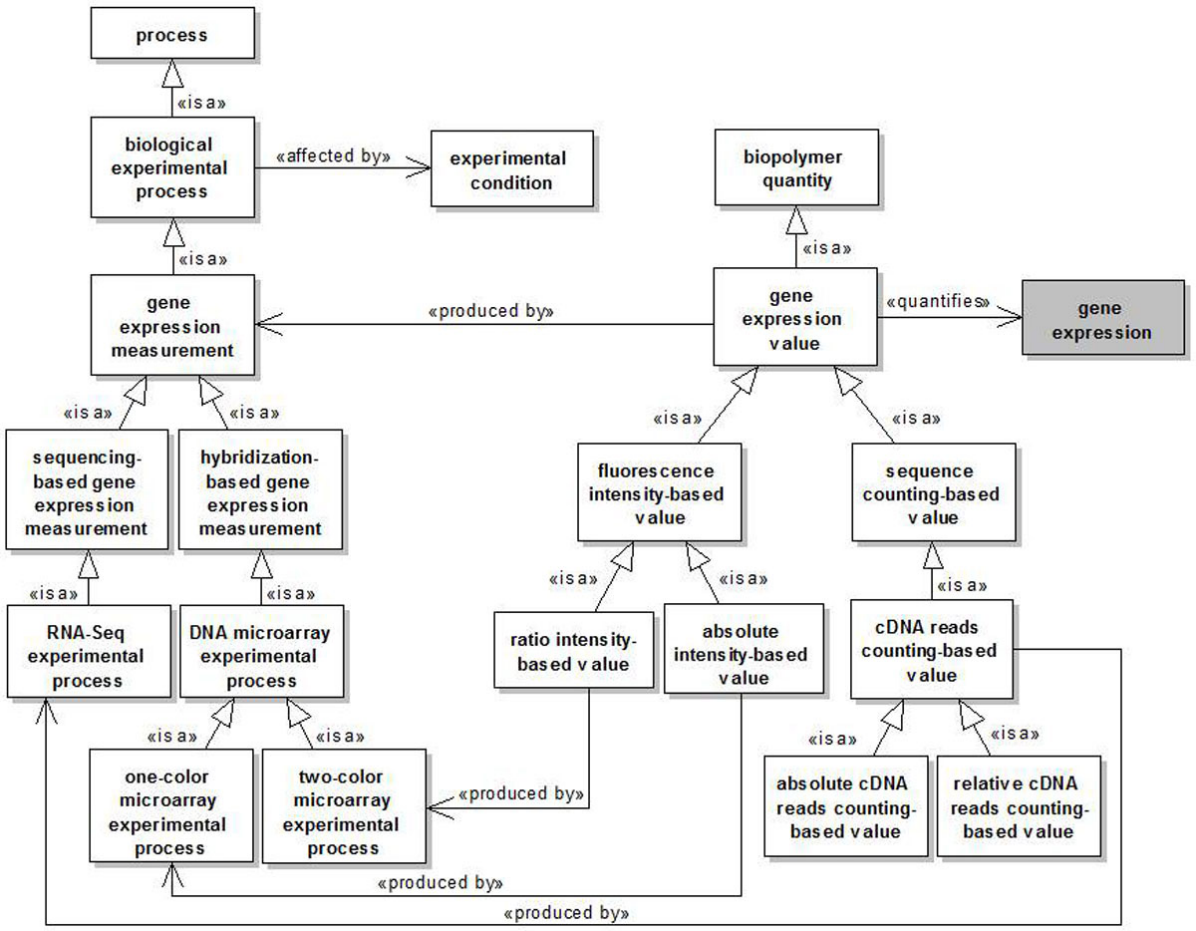
**Gene expression measurement process**. A named rectangle represents a concept. A white rectangle represents a concept introduced in our ontology, while a gray rectangle represents a concept reused from another ontology. A solid line with a stick arrowhead at its end represents a relationship between two concepts. A solid line with a hollow triangle as an arrowhead represents an *is a *relationship.

**Figure 4:**
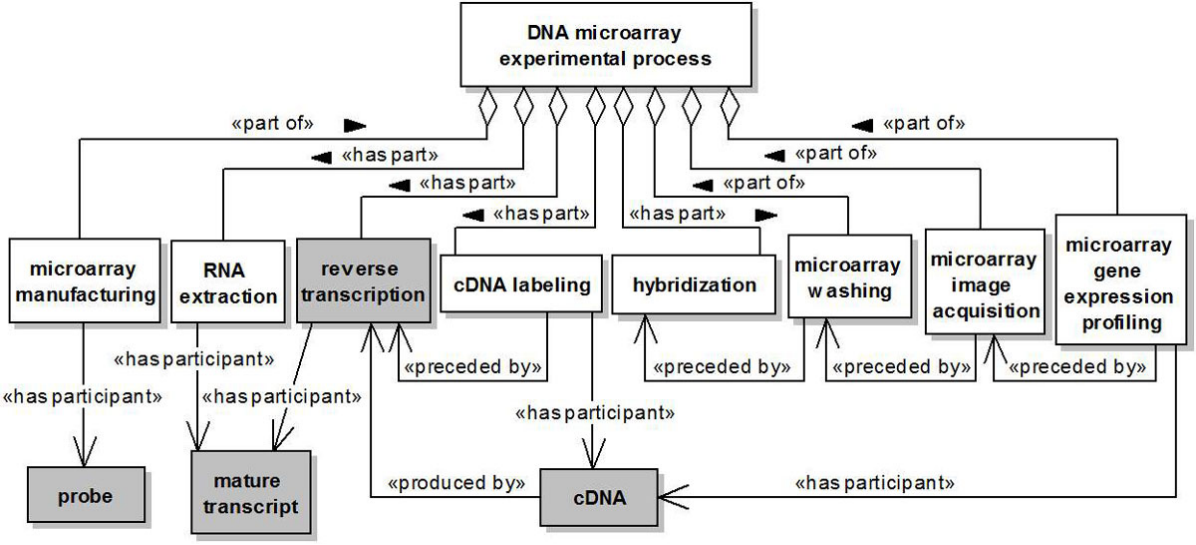
**DNA microarray experimental process**. A named rectangle represents a concept. A white rectangle represents a concept introduced in our ontology, while a gray rectangle represents a concept reused from another ontology. A solid line with a stick arrowhead at its end represents a relationship between two concepts. A solid line with a hollow diamond at its end represents either a *part of *or a *has part *relationship.

**Figure 5:**
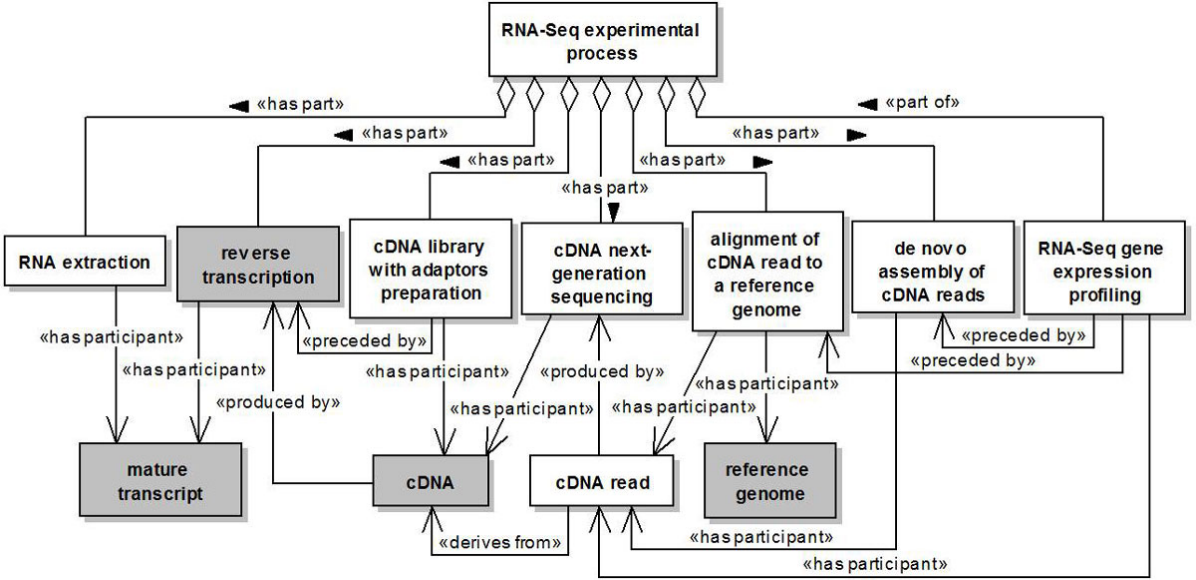
**RNA-Seq experimental process**. A named rectangle represents a concept. A white rectangle represents a concept introduced in our ontology, while a gray rectangle represents a concept reused from another ontology. A solid line with a stick arrowhead at its end represents a relationship between two concepts. A solid line with a hollow diamond at its end represents either a *part of *or a *has part *relationship.

All classes (concepts) defined in our reference ontology are subclasses of the general OWL class *Thing*. Figures 2, 3, 4 and 5 present the concepts and relationships defined as part of the ontology through UML class diagrams. Not all concepts and relationships are graphically presented using UML, only those which were considered more relevant to the application of the developed methodology to the proposed integration scenarios. A concept in our ontology is represented by a UML class. The relationships between concepts are presented through a notation similar to the one proposed in [70]. The subsumption relation of OWL is presented as a UML generalization stereotyped as is a. The *part *of relation and its inverse (*has part*) are presented as a UML shared aggregation stereotyped as *part of *and *has part*, respectively. The other relations are presented as stereotyped UML associations.

Particularly, the concepts reused from the Gene Ontology represent biological processes, such as *gene expression, reverse transcription *and *translation*, while the concepts reused from Sequence Ontology represent *"things"*, such as *DNA, mature transcript *and *protein*. Most of the relationships used to relate these and other concepts were reused from the OBO Relation Ontology [71,72], except for the *produced by, affected by *and *quantifies *relations, which we have introduced in our ontology. The *produced by *relation was defined as a relation between a process and an entity produced by this process. The *affected by *relation was defined as a relation between an experimental process and an experimental condition capable of affecting this process. Finally, the *quantifies *relation was defined as a relation between a biopolymer quantity and the biopolymer which it quantifies.

Figure 2 presents the concepts associated to gene expression.

The class *gene expression *represents a biological process (class *biological process*) by which the information codified by a gene is used to synthesize functional gene products, i.e., proteins and functional RNAs. The classes *transcription, RNA processing, translation *and *protein maturation *represent different subprocesses involved in gene expression. These subprocesses are related to the class *gene expression *through *part of *relations. The relationship *preceded by *defined between the classes *RNA processing and transcription *indicates that the occurence of RNA processing is preceded by the occurence of transcription. This relationship is also defined between the classes *translation *and *RNA processing*, and between the classes *protein maturation *and *translation*.

The class *transcription *represents the production (relationship *produced by*) of a primary transcript (class *primary transcript*) from the information codified (relationship *has participant*) by a gene (class *gene*). The class *RNA processing *represents the occurence of modifications in a primary transcript (*relationship has participant*), such as polyadenylation and splicing. The *class mature transcript *represents the result of this subprocess (relationship *produced by*). There are two subtypes of mature transcript: non-coding RNA (class *n*c*RNA*) and messenger RNA (class *mRNA*). The class *translation *represents the production (relationship *produced by*) of a protein (class *protein*) from the information codified in a mRNA molecule (relationship has *participant*). Finally, the class *protein maturation *represents the occurence of chemical modifications in a protein (relationship *has participant*), leading to the attainment of its full functional capacity.

Figure 3 presents the concepts associated to the gene expression measurement process.

The class *biological experimental process *represents a general biological experimental process. The class *experimental condition *represents any experimental condition that can affect a biological experimental process (relationship *affected by*). The class *gene expression measurement *represents a specific experimental process whose objective is to quantify the functional products, i.e., functional RNAs and proteins, produced by a gene as result of its expression. In the context of this work, this process can be realized through a hibridization-based or sequencing-based approach (subclasses *hybridization-based gene expression measurement *and *sequencing-based gene expression measurement*). The class *DNA microarray experimental process *specializes the class *hybridization-based gene expression measurement*. Basically, DNA microarray experimental process represents the process by which DNA probes attached to the surface of microarray chips are used to hybridize with a labeled cDNA sample, in order to quantify functional gene products. The classes *one-color microarray experimental process *and *two-color microarray experimental process *also specialize *DNA microarray experimental process*. The class *one-color microarray experimental process *represents a specific DNA microarray experimental process in which the biological samples of interest are labeled with the same fluorescent dye and hybridized in different arrays. Conversely, the class *two-color microarray experimental process *represents a specific DNA microarray experimental process in which the biological samples of interest are labeled with different fluorescent dyes and hybridized in the same array. Finally, the class RNA-Seq *experimental process *specializes *sequencing-based gene expression measurement*. Basically, RNA-Seq experimental process represents the process by which cDNA molecules produced from a biological sample of interest are sequenced through next-generation sequencing technologies and quantified.

The class *gene expression value *represents a value obtained by quantifying the functional products produced by a gene as a result of its expression (relationship *quantifies*). The classes *fluorescence intensity-based value *and *sequence counting-based value *specialize *gene expression value*. Additionally, the classes *absolute intensity-based value *and *ratio intensity-based value *specialize *fluorescence intensity-based value *to represent the values produced by one-color and two-color microarray experimental processes (relationships *produced by *), respectively. Specifically, the class *absolute intensity-based value *represents a value that quantifies the expression of a gene under a unique experimental condition, while the class *ratio intensity-based value *represents the ratio between the levels of expression of a gene under two different experimental conditions. The class *cDNA reads counting-based value *specializes *sequence counting-based value. cDNA reads counting-based value *represents a value produced by a RNA-Seq experimental process (relationship *produced by *) to quantify the expression of a gene. The classes *absolute cDNA reads counting-based value *and *relative cDNA reads counting-based value *specialize *cDNA reads counting-based value *to represent that the value obtained from a RNA-Seq experimental process may be based on the absolute number of cDNA reads or on a relative value, respectively.

Figure 4 presents the concepts associated to a DNA microarray experimental process.

The class *DNA microarray experimental process *represents a hybridization-based experimental process aiming at quantifying the functional products generated by a gene as result of its expression. The classes *microarray manufacturing, microarray washing, microarray image acquisition *and *microarray gene expression profiling *represent different subprocesses of a DNA microarray experiment. These subprocesses are associated to the class *DNA microarray experimental process *through *part of *relations, since they only occur as part of a DNA microarray experimental process. The classes *RNA extraction, reverse transcription, cDNA labeling *and *hybridization *also represent subprocesses of a DNA microarray experiment. However, these subprocesses are associated to the class *DNA microarray experimental process *through *has part *relations, since they can also occur as part of other biological or experimental processes.

The class *microarray manufacturing *represents the process of manufacturing a microarray chip with probes of interest (relationship *has participant *). The class *probe *represents fragments of DNA used in the microarray manufacturing process. The class *RNA extraction *represents the process by which functional RNA molecules (class *mature transcript *) are extracted from cells or tissues of interest (relationship *has participant *). The class *reverse transcription *represents the biological process by which a complementary DNA (cDNA) molecule is produced (relationship *produced by *) from a mature transcript (relationship *has participant *). The class *cDNA labeling *represents the process by which cDNA molecules (class *cDNA*) are labeled with fluorescent dyes (relationship *has participant *). The class *hybridization *represents the biological process by which two or more complementary nucleic acids establish non-covalent interactions, pairing each other. Specifically, in a DNA microarray experiment, the process of hybridization occurs between a DNA fragment and a cDNA molecule. The class *microarray washing *represents the washing of a microarray chip aiming at removing cDNA molecules that were not hybridized to the chip probes. The class *microarray image acquisition *represents the process of obtaining a microarray image. A microarray image is produced by exciting the labeled cDNA molecules with a laser and scanning the microarray chip in order to measure the emission of these molecules. Finally, the class *microarray gene expression profiling *represents the measurement of gene expression levels (hybridized cDNA) by quantifying the fluorescence intensities contained in a microarray image (relationship *has participant *).

The relationship *preceded by *defined between the classes *cDNA labeling *and *reverse transcription *indicates that the process of cDNA labeling is preceded by the occurence of reverse transcription. This relationship is also defined between the classes *microarray washing *and *hybridization, microarray image acquisition *and *microarray washing*, and between the classes *microarray gene expression profiling *and *microarray image acquisition*.

Figure 5 presents the concepts associated to a RNA-Seq experimental process.

The class *RNA-Seq experimental process *represents a sequencing-based experimental process aiming at quantifying the functional products generated by a gene as result of its expression. The class *RNA-Seq gene expression profiling *represents a subprocess of a RNA-Seq experiment. This subprocess is associated with *RNA-Seq experimental process *through a *part of *relation, since it only occurs as part of a RNA-Seq experimental process. The classes *RNA extraction, reverse transcription, cDNA library with adaptors preparation, cDNA next-generation sequencing, alignment of cDNA read to a reference genome *and *de novo assembly of cDNA reads *also represent subprocesses of a RNA-Seq experiment. However, these subprocesses are associated with *RNA-Seq experimental process *through *has part *relations, since they can also occur as part of other biological or experimental processes.

The class *cDNA library with adaptors preparation *represents the preparation of a cDNA library containing cDNA molecules (relationship *has participant *) with adaptors. The class *cDNA next-generation sequencing *represents the process of sequencing a cDNA molecule (relationship *has participant *) through next-generation sequencing technologies in order to produce cDNA reads (relationship *produced by *). The class *cDNA read *represents a cDNA sequence fragment obtained as a result of a sequencing experiment (relationship *derives from*). The class *reference genome *represents a standard collection of sequences for a given organism and genome assembly. The class *alignment of cDNA read to a reference genome *represents the process of aligning a cDNA read to a reference genome (relationship *has participant *). The class *de novo assembly of cDNA reads*, in turn, represents the process of assembling cDNA reads (relationship *has participant *) to create a transcriptome without the aid of a reference genome. The classes *alignment of cDNA read to a reference genome *and *de novo assembly of cDNA reads *represent alternative processes of a RNA-Seq experiment (choice relationship not depicted in Figure 5). Finally, the class *RNA-Seq gene expression profiling *represents the measurement of gene expression levels based on the counting of cDNA reads (relationship *has participant *).

The relationship *preceded by *defined between the classes *cDNA library with adaptors preparation *and *reverse transcription *indicates that preparing a cDNA library with adaptors is preceded by the occurrence of reverse transcription. This relationship is also defined between the class *RNA-Seq gene expression profiling *and the classes *alignment of cDNA read to a reference genome *and *de novo assembly of cDNA reads*. However, since *alignment of cDNA read to a reference genome *and *de novo assembly of cDNA reads *represent alternative processes of a RNA-Seq experiment, gene expression profiling is preceded by the occurrence of either one of these processes, not both (choice relationship not depicted in Figure 5 either).

The complete OWL specification of the Gene Expression Ontology, including the definition of a SAGE experimental process, can be found in a supplementary material (see Additional File 1: OWL specification of the Gene Expression Ontology).

### Connector development methodology

A methodology consists of a collection of systematic procedures or guidelines used to produce an intended set of artifacts (e.g., documents, models and code) to represent the different elements of a target application domain. Thus, we defined a set of activities and associated guidelines that prescribe how the development of a connector *C *to integrate tools *T_A _*and *T_B _*or to integrate a tool *T_A _*to a data source *D *should be carried out:

1. *Identification of main functionalities*. This activity aims simply at identifying the main functionalities provided by the tools being integrated. We should develop a functional specification listing the main functionalities F_*A*1_, F_*A*2_, ..., F*_AN _*and F_*B*1_, F_*B*2_, ..., F_*BM *_of tools *T*_*A *_and *T*_*B*_, respectively. This abstract specification both provides a better understanting of the services provided by each tool and serves as a starting point for the identification of possible integration scenarios. Considering the integration of a data source *D *to a tool *T_A_*, only the functionalities of *T_A _*should be identified.

2. *Integration scenario initial description*. Since each tool being integrated provides different sets of functionalities and since these sets of functionalities can possibly be combined and used in many different ways, we need to delimit a target integration scenario. So, this activity aims at providing an initial description of this target scenario. Such a description can be obtained in three steps. First, we should select among the functionalities previously identified a set of interest functionalities for the target scenario. Then, we should identify any new functionality that should be provided as part of the integration scenario. Such functionality will be provided by the connector under development itself. Together, both sets of funcionalities form the set of relevant functionalities for the target scenario. Finally, we should provide a general textual description of this scenario. Such a description must include all relevant functionalities identified.

3. *Integration scenario detailed description*. After the initial description of the integration scenario, we need to detail this scenario. So, this activity aims at providing a detailed description of the target integration scenario through the development of an activity model and a use case model. These models can be obtained through the development of UML activity and use case diagrams [66,67], respectively. The development of an activity model aims at capturing the order in which the relevant functionalities are executed and the functional entities (tool or connector) responsible for their execution. The development of a use case model aims at capturing additional information regarding the execution of the relevant functionalities through the description of the interactions between the functionality users and the entities responsible for their execution.

An activity diagram describes a coordinated execution of a sequence of activities performed by one or more functional entities. Each relevant functionality should be mapped to a corresponding activity. Activities can be organised into swimlanes, which represent responsibility zones. Thus, swimlanes, one for each entity, should be used to associate each activity to the entity responsible for its execution. The execution ordering of the identified activities should be established based on the integration scenario initial description.

A use case diagram describes an interaction scenario between a set of users and a functional entity. A use case diagram consists of a set of actors, use cases and their relationships. A use case represents a unit of functionality comprising sequences of actions that the functional entity perform to produce an observable result to one or more outside interactors, called actors. An actor represents a role that a user plays with respect to the functional entity. Use cases are identified based on the developed activity model. In general, each identified activity should be mapped to a single use case. However, multiple (fine-grained) activities could also be mapped to a single use case. Each identified use case should be associated to either one of the tools involved in the integration or the connector itself. This association is directly obtained from the activity diagram swimlanes. Actors and their association to use cases should be identified based on the integration scenario initial description.

A detailed description of selected use cases should complement the use case diagram. Such a description consists of a textual description of the main aspects associated to the use case in a table-like format. For each use case, the following information should be provided: use case purpose, list of associated actors, numbered account of the interactions initiated by the associated actors and the corresponding functional entity response. These interactions should be described in a typical or normal course of events, which does not preclude the description of alternative courses of events either. The selection of use cases to be described is based on the activity diagram. Only use cases created based on activities directly related to the interactions between a tool or a data source and the connector should be described.

As a final and complementary step of the integration scenario detailed description, we should provide an architectural description of the scenario to facilitate the visualization of the different roles played by the involved entities. In order to produce such a description we can use an ADL or simply represent graphically the different architectural elements (tools, data sources and connectors) and their relationships using an *ad hoc *notation. At this point, we can structure (refine) a complex (composite) connector as an integrated set of simple connectors. This can be accomplished through the assignment of different use cases to different (simple) connectors.

4. *Interest data detailed description*. This activity aims at providing a detailed description of the integration interest data. Interest data include data either consumed or produced by a connector. Such information can be typically obtained from the use case detailed descriptions. However, other sources can be used to provide additional information, such as user manuals, help entries, etc. For each functionality F*_i _*previously associated to a use case, we should describe in a table format each data item either consumed or produced.

Two separate tables should be created for each connector: one to describe data items that are consumed and one to describe data items that are produced. For each identified data item, a new row should be added to the table. A separate column should be used to describe the data item identification, the data item semantic description and the data item syntactic description. The data semantic description contains a textual description of the intended meaning of the data item. The data syntactic description contains all information needed to concretely represent the data, including the data storage medium (e.g., text file, binary file, database entry, etc), encoding format (e.g., ASCII, Unicode, etc), content type (e.g., integer, float, string, etc) and cardinality (e.g., single entry, array, matrix, etc).

5. *Interest data conceptual modelling*. After the detailed description of the interest data, we should develop conceptual models to represent such information at a high abstraction level. Two conceptual models should be developed for each (simple) connector using UML class diagram [66,67]: one to represent the data consumed by the connector and another to represent the data produced by the connector.

Each conceptual model should formalize the concepts and relationships identified in the interest data detailed description. Each identified data item should be mapped to a corresponding concept (UML class) in the concept model. Additionally, any identified relationship between data items should be mapped to a corresponding relationship between concepts. Typical relationships include UML association and aggregation.

6. *Reference ontology mapping*. After creating the conceptual models of interest data, we should map the concepts present in these models to concepts present in the reference ontology. So, this activity aims at identifying the correspondence between concepts representing either consumed or produced data items and concepts from the reference ontology.

**Figure 6:**
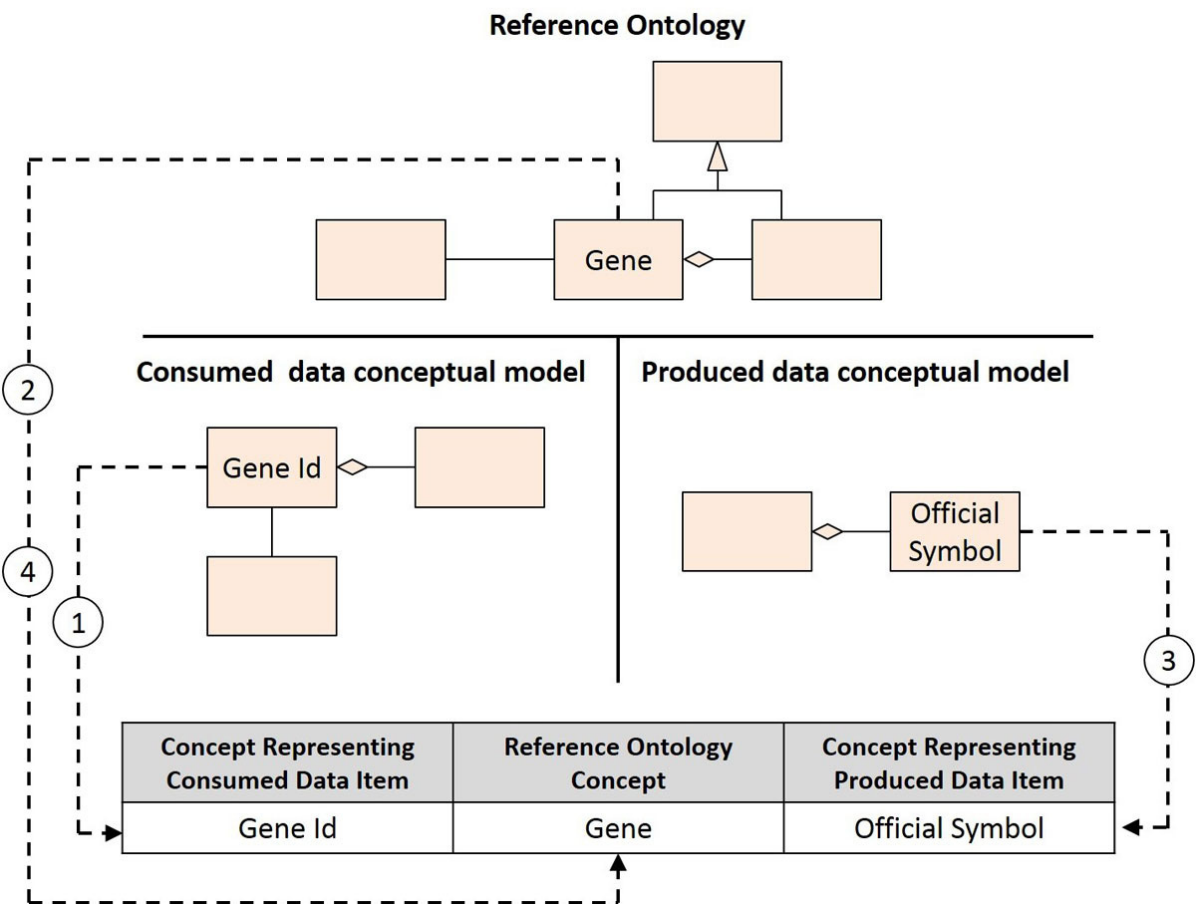
**Equivalence table creation**. Concepts representing consumed and produced data items are mapped to a reference ontology concept. 1) The concept *Gene Id *is added to the consumed data item concept column; 2) An equivalence is identified between the *Gene Id *concept and the reference ontology concept of *Gene*; 3) The concept *Official Symbol *is added to the produced data item concept column; 4) An equivalence is identified between the *Official Symbol *concept and the reference ontology concept of *Gene*; rows representing consumed and produced data items are merged.

The mapping can be accomplished through the construction of an equivalence table for each (simple) connector. This table contains three columns: the first column contains concepts representing consumed data items; the second column contains concepts present in the reference ontology; and, the third column contains concepts representing produced data items. First, we should add one row for each concept representing an identified consumed data item. Then, we should identify the equivalence between each one of these concepts and a corresponding concept present in the reference ontology. Whenever such equivalence can be identified, the corresponding reference ontology concept should be added to the appropriate row. Finally, we should also add one row for each concept representing an identified produced data item and then identify the equivalence between each one of these concepts and a corresponding concept present in the reference ontology. Once again, whenever such equivalence can be identified, the corresponding reference ontology concept should be added to the appropriate row. In case the same concept appears repeatedly in different rows, it should be merged into a single row. Figure 6 illustrates the construction of an equivalence table.

At the end of the equivalence table construction, ideally we should have mapped each concept representing a consumed data item to a corresponding concept from the reference ontology, which also corresponds to a concept representing a produced data item. However, situations in which (at first) partial mappings are obtained are quite common and should be dealt with accordingly.

Three cases in which a partial mapping is obtained can be identified: 1) a consumed data item is simply not used by the connector to produce an output; 2) a consumed data item may represent part of the information used by the connector to produce an output; and 3) a concept representing a produced item may be derived from the input data. Since, in case 1, the data is not used to produce an output, the lack of correspondence to a reference ontology concept is meaningless for the problem at hand, so the complete mapping is not required. In cases 2 and 3, the definition of of semantic equivalence rules is required (see activity *Grounding and semantic transformation definition*.).

After the specification of semantic equivalence rules, table entries should be merged or split accordingly. Once such correspondences can be established for all consumed and produced data items, except for unused data items, we can assert that semantic integration can be obtained.

7. *Grounding and semantic transformation definition*. This activity aims at describing the mapping between syntactic and semantic data descriptions. Additionally, this activity also aims at describing any possible semantic mapping transformation that might be required in order to establish equivalent relations between concepts representing produced and/or consumed data items and concepts from the reference ontology.

The mapping between syntactic and semantic data descriptions is often called grounding in the literature [73]. Grounding is required because frequently the same semantic information can be represented differently at a syntactic level (e.g., different formats for date representation). Grounding can be specified by means of two complementary operations: *lifting*, which is used to interpret semantic data from a (concrete) syntactic representation, and *lowering*, which is used to create a (concrete) syntactic representation from semantic data. In the context of this work, lifting and lowering operations should be specified textually for each consumed and produced data item. This specification should be based on the *Interest data detailed description *and *Reference ontology mapping *activities and serves as basis for the *Connector implementation *activity.

During the specification of the lifting and lowering operations, we need to identify and represent any semantic transformation between consumed data conceptual model elements and reference ontology elements as well as between reference ontology elements and produced data conceptual model elements that might be necessary. The need for such semantic transformation might have been identified during the *Reference ontology mapping *activity. In such case, we need to formally represent either mathematically or through a series of inference rules how a produced data conceptual model element can be derived from a reference ontology element or from a set of consumed data conceptual model elements, thus creating an equivalence relation between all these concepts. Once this equivalency is defined, the equivalence table developed at the *Reference ontology mapping *activity should be updated.

8. *Access policy identification*. This activity aims at providing a textual description of the integration target object (tool or data source) access policy. This description specifies how and where the functionalities of the integration target object can be accessed by the connector under development.

The access policy description should include the identification of the mechanism to be used to either access consumed and/or produced data (e.g., SQL query mechanism (relational databases), file input/output (binary or text files), etc) or provide (automatic) transfer of data and control to the integration target object (e.g., procedure call, Unix/Linux pipes, TCP/UDP communication sockets, HTTP connections, etc). Automatic transfer of data and control is usually available only whenever an API describing the integration target object functionalities exists.

The access policy description should also include information regarding the physical and logical location (e.g., local directory, file name, database name, URL, IP address, etc) of the integration target object, as well as any existing access restriction to the object (e.g., public, protected or private access with authorization). This information can be usually obtained from user manuals, help pages, API documentation, etc.

9. *Connector implementation*. This activity aims at implementing the connector in a systematic way. There is no restriction on the choice of implementation language to be used. The connector's developer is free to use any language she or he sees suitable for this task. However, some of the guidelines provided below take into account the use of an object-oriented programming language.

The functionalities of each connector should be structured into four functional blocks: 1) *Data Input Processing*, which is responsible for obtaining the input data according to the specified data input format and producing an object-oriented representation of the data; 2) *Lifting*, which is responsible for transforming the previously obtained object-oriented data representation into a reference (canonical) object-oriented representation of the data; 3) *Lowering*, which is responsible for transforming the previously obtained canonical representation into an object-oriented representation of the data suitable for output; and 4) *Data Output Processing*, which is responsible for producing the desired output according to the specified data format based on the previously obtained object-oriented representation of the data.

The *Lifting *functional blocks is also responsible for implementing any required semantic transformation of the concepts according to the equivalence relations specified in the *Grounding and semantic transformation definition *activity. The *Data Output Processing *functional block is also responsible for accessing the target tool or data source being integrated and realizing the transfer of data and/or control as specified in the *Access policy identification *activity.

**Figure 7:**
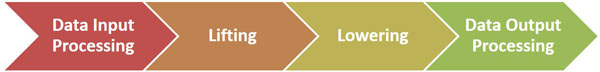
**Connector implementation functional blocks**. An arrow represents an implementation functional block. The left to right serial disposition of the arrows indicates the order in which each functional block should be executed, starting at the Data Input Processing block.

The requirements for the implementation of each functional block can be identified based on the information produced by the *Interest data detailed description, Grounding and semantic transformation definition *and *Access policy identification *activities. The execution of the functional blocks should be carried out serially according to the order in which they have been defined. Figure 7 illustrates the timeline execution of a connector's implementation functional blocks.

The connector can be implemented completely independent from tool *T_A _*or as an integral part of this tool. The independent connector implementation is simpler to be implemented since no changes are required to tool *T_A_*. However, this implementation choice relies on the user to explicitly execute the connector. The integral part connector implementation enables the connector to be automatically executed from tool *T_A _*integrated interface. However, this implementation choice relies on the availability of the source code of this tool.

### Methodology application

We applied the proposed methodology in the development of three integration scenarios for the analysis of different types of gene expression data.

In the first scenario, two-color *Plasmodium vivax *microarray data were normalized using RGUI. In order to facilitate multiple condition normalization by RGUI, different two-color microarray data should be combined into a single multi-column dataset. Normalized microarray data was then used by RGUI itself for the identification of differentially expressed genes. In the sequel, KEGG *Plasmodium vivax *pathways were analysed using KEGG Mapper - Search&Color Pathway. This tool was used to identify which parts of pathways were associated with the differential gene expression analysis provided by RGUI, highlighting up and down regulation according to a color scheme.

**Figure 8:**
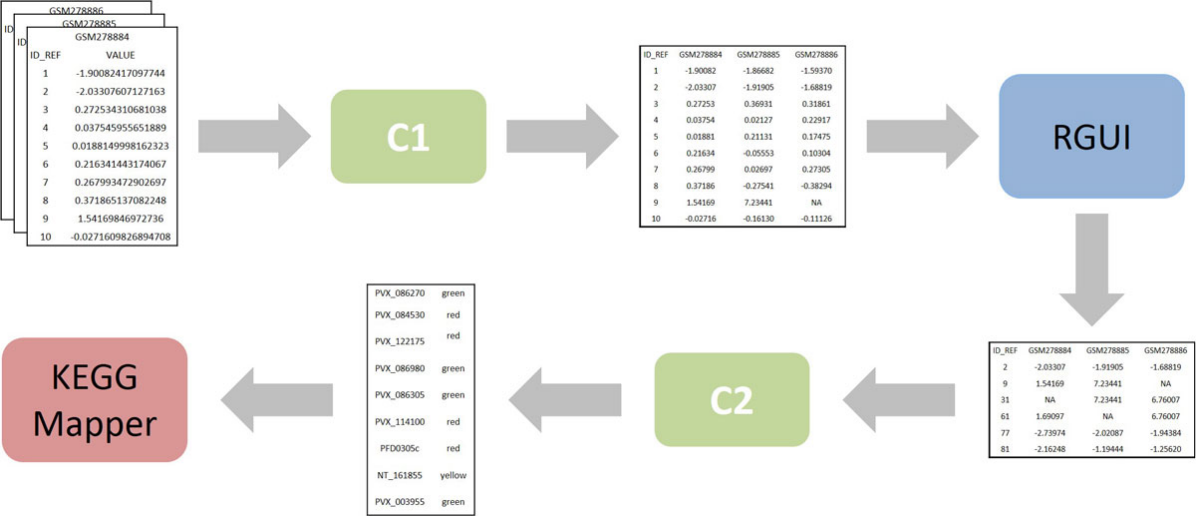
**Integration architecture of two-color microarray data to tools RGUI and KEGG Mapper - Search&Color Pathway**. A rectangle represents a data source, while a rectangle with rounded corners represents either a tool or a connector. An one-way arrow represents a directed flow of data and/or control.

Figure 8 illustrates the architecture of our first integration scenario with focus on the flow of data. Two connectors were developed to integrate two-color microarray data to RGUI and KEGG Mapper. Connector *C1 *groups separate two-color microarray data into a single two-color microarray dataset to facilitate normalization by RGUI, while connector *C2 *processes RGUI output data, so they can be used by KEGG Mapper.

Different files were used to store two-color microarray data (SOFT format), each representing a separate experimental condition. So basically, connector *C1 *was responsible for reading these files and for producing a single (multi-column) file containing all data to facilitate further analysis activities. From a semantic point of view, the construction of the equivalence table was straightforward since the same set of concepts was used to represent data items both consumed and produced by the connector. These concepts were also directly mapped to the concepts of the reference ontology. Thus, no transformation was required.

Connector *C1 *was implemented as a separate Java application. Its different functional blocks were implemented as follows. The *Data Input Processing *functional block reads a number of files containing two-color microarray data and stores these data as lists of strings (one list for each file). Then, the *Lifting *functional block transforms the lists of strings into three separate lists, containing instances of genes, experimental conditions and multiple lists of ratio intensity-based values. Next, the *Lowering *functional block transforms all the previous lists into a single list of strings. Each string represents the concatenation (tab-delimited) of the different instances of the elements identified in the *Lifting *functional block. Finally, the *Data Output Processing *functional block simply writes the list of strings previously produced into a single text file (one string per line). This general strategy has been similarly adopted in the implementation of all connectors in the context of this work.

Connector *C1 *was also designed to provide both manual and automatic transfer of control to RGUI (parameter-based selection). The automatic integration of the connector with RGUI was provided by the RServe API. Since the connector is capable of providing both forms of transfer of control to RGUI, the automatic integration was implemented independently of the *Data Output Processing *functional block in order to maintain a sound implementation structure. Nevertheless, the interaction with RGUI takes place after data output processing is complete. Detailed information regarding connector *C1 *implementation can be obtained in a supplementary material (see Additional File 2: Connectors *C1 *and *C2 *Implementation).

Finally, we have also developed an API, called Gene Expression Library Class (GELC), to facilitate the implementation of any semantic-based application in the domain. This API contains a number of Java classes, each corresponding to a different concept of our reference ontology. Examples of classes contained in the GELC API include *AbsoluteIntensityBasedValue, CDNARead, ExperimentalCondition, Gene, RatioIntensityBasedValue, SAGETag*, etc. All connectors were implemented using the GELC API in the context of this work. The GELC API binary code and documentation is also available as supplementary material (see Additional File 3: GELC API).

RGUI produced as output a single file containing the result of the differential gene expression analysis. These data were processed directly by connector *C2 *in order to produce an input suitable for KEGG Mapper - Search&Color Pathway. Connector *C2 *also received two user-provided (auxiliary) inputs, viz., *i) *the identification of the experimental condition whose expression values should be considered for further analysis; and *ii) *the value of a gene expression threshold that was used in the gene expression (regulation) classification (upregulated, downregulated and undefined). As a result, connector *C2 *produced as output a list of genes and their respective color information, viz., red, green or yellow, to be used as input by KEGG Mapper.

The semantical mapping between concepts representing either consumed or produced data items and concepts from the reference ontology for connector *C2 *was not as direct as for connector *C1*. There were cases where no direct association between a concept representing a consumed data item and a concept representing a produced data item were identified. So, two equivalence relations had to be created for those cases. The first equivalence relation was straightforward and was created to associate an instance of the concept *experiment-specific gene identifier *with a possible instance of the concept *KEGG identifier*. Although both concepts were associated to the reference ontology concept of *gene*, not all instances of *experiment-specific gene identifier *have corresponding instances of *KEGG identifier*. The second equivalence relation was created to associate the instances of the concepts of *experimental condition, ratio intensity-based value *and *gene expression threshold *with an instance of the concept of *ratio intensity-based value*. Although quite simple in principle, the definition of this equivalence relation was quite elaborated. It involved the definition of a mapping over instances of *ratio intensity-based value *from the real numbers domain to the up, down and undefined regulation range according to a (user-provided) instance of *gene expression threshold*.

Connector *C2 *was also implemented as a separate Java application. This connector provided only manual transfer of control to KEGG Mapper - Search&Color Pathway. Once the equivalence relations were defined, the specification and implementation of the grounding operations was straightforward. Additionally, the transformation of instances of the concept *experiment-specific gene identifier *to instances of the concept *KEGG identifier *was carried out using a platform mapping file (SOFT format), also provided as input to connector *C2*. This mapping is usually carried out in two steps: 1) from experiment-specific gene identifier to official gene identifier; and 2) from official gene identifier to KEGG identifier. However, in the specific case of *Plasmodium vivax*, each KEGG identifier corresponds directly to its official gene identifier. In the implementation of the *Lowering *functional block instances of the concept *ratio intensity-based value*, viz., *upregulated, downregulated *and *undefined*, were mapped respectively to the red, green and yellow KEGG Mapper - Search&Color Pathway color schema. Detailed information regarding connector *C2 *implementation can also be obtained in a supplementary material (see Additional File 2: Connectors *C1 *and *C2 *Implementation).

The second scenario consisted of the integration of tools DMV and TMev in order to clusterize *Sulfolobus solfataricus *RNA-Seq data. This scenario was inspired by the increasing popularity of next-generation sequencing platforms for gene expression. Despite the benefits of storing the actual reads in RNA-Seq databases, these raw data files provide little support for high-level gene expression analysis. So, they were transformed into a numeric representation to be filtered using DMV according to some user defined criteria. Next, DMV output was used as input data for clustering using TMev. However, DMV output data must be normalized to account for different library sizes (total of sequenced reads) before clusterization by TMev.

**Figure 9:**
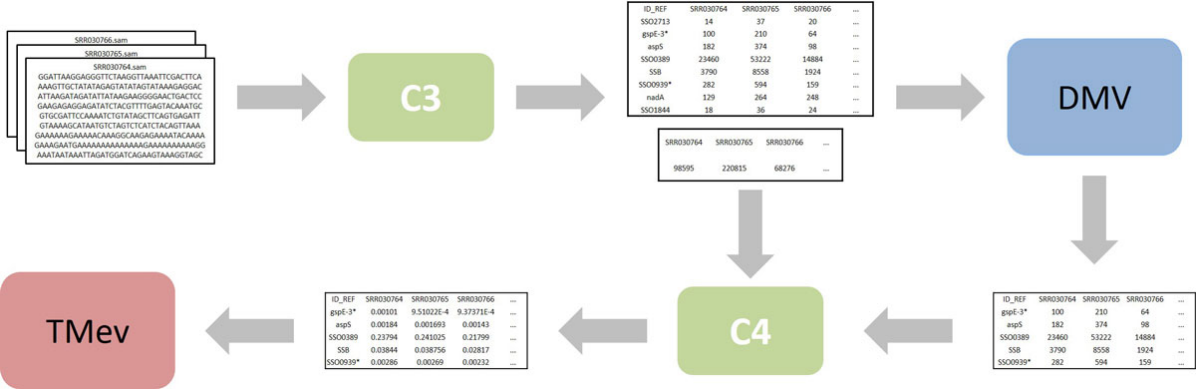
**Integration architecture of RNA-Seq data to tools DMV and TMev**. A rectangle represents a data source, while a rectangle with rounded corners represents either a tool or a connector. An one-way arrow represents a directed flow of data and/or control.

Figure 9 illustrates the architecture of our second integration scenario with focus on the flow of data. Two connectors were developed to integrate RNA-Seq data to DMV and TMev. Connector *C3 *transforms RNA-Seq data, so they can be filtered by DMV, while connector *C4 *normalizes DMV filtered data, so they can be clusterized by TMev.

Different files were used to store aligned RNA-Seq data (SAM format), each representing a different experimental condition. RNA-Seq data was then transformed into a numeric representation in a three-step process. The first step was the identification of known transcribed genes (represented by cDNA reads) using the *Sulfolobus solfataricus *genome description (GFF format) and replacing them in each source file with the associated gene identifiers (annotation). In case no match was found, the associated cDNA read was removed from the source file. The second step was the counting of the total number of annotated genes present in each source file. The result was then stored in a text file (tab-delimited format - one column for each experimental condition). The third step was the counting of the number that each annotated gene appears in each experimental condition. Once again, the result was stored in a text file (tab-delimited format - one column for each experimental condition and one row for each annotated gene identifier).

**Figure 10:**
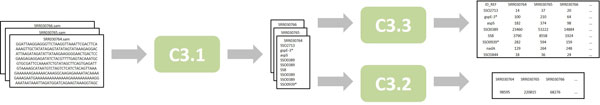
**Connector C3 architecture**. A rectangle represents a data source, while a rectangle with rounded corners represents a simple connector. An one-way arrow represents a directed flow of data.

Connector *C3 *was created to integrate RNA-Seq data to DMV. *C3 *represents a composite connector consisting of three simple connectors: *C3.1, C3.2 *and *C3.3*, which are responsible for performing steps one to three of the aforementioned transformation process, respectively. Figure 10 shows the internal architecture of connector *C3*, focusing on the flow of data between connectors.

The semantical mapping between concepts representing either consumed or produced data items and concepts from the reference ontology for connector *C3 *was not straightforward. In several cases, there was no direct association between a concept representing a consumed data item and a concept representing a produced data item. So, equivalence relations had to be created for those cases. For example, in connector *C3.1*, an equivalence relation was created to associate an instance of the concept *cDNA read *with an instance of the concept *gene *(inferred from the reference ontology); in connector *C3.2*, an equivalence relation was created to associate an instance of the concept *gene *with an instance of the concept *absolute cDNA reads counting-based value *(the total number of cDNA reads represents the absolute number of instances of all genes observed in a RNA-Seq data file obtained according to a particular experimental condition); finally, in connector *C3.3*, another equivalence relation was created to associate instances of the concepts *gene *and *experimental condition *with an instance of the concept *absolute cDNA reads counting-based value *(the number of cDNA reads represents the absolute number of instances of a particular gene observed in a RNA-Seq data file obtained according to a particular experimental condition).

Once the equivalence relations were defined, the specification and implementation of grounding operations was carried out based on these definitions. Connectors *C3.1, C3.2 *and *C3.3 *were each implemented as a separate Java application. Thus, each connector can be executed and (re)used independently. These simple connectors were then composed to form connector *C3*, which is responsible for controlling the ordering in which the simple connectors are executed, viz., first *C3.1*, then *C3.2 *and finally *C3.3*. Although connectors *C3.2 *and *C3.3 *can be executed in any order (even concurrently), we have chosen that specific sequencing because performance is not an issue in the scope of this work. Connector *C3 *as a whole was designed to provide only manual transfer of control to DMV, since this tool does not provide an API for automatic interaction from a third-party application.

Data output from DMV must be normalized before they can be clusterized by TMev to account for different library sizes. Normalization was carried out by connector *C4 *by dividing the number that each annotated gene appears in each experimental condition by the total number of annotated genes present in each source file. These normalized data produced by connector *C4 *were then used as input by TMev.

Similarly to connector *C3*, the semantical mapping between concepts representing either consumed or produced data items and concepts from the reference ontology for connector *C4 *was not straightforward either. So, an equivalence relation was defined to associate two instances of the concept of *absolute cDNA reads counting-based value *with one instance of the concept of *relative cDNA reads counting-based value *(relative cDNA reads counting-based value represents the normalization of the absolute number of instances of a particular gene by the absolute number of instances of all genes according to a particular experimental condition).

Connector *C4 *was also implemented as a separate Java application. This connector provided only manual transfer of control to TMev, since this tool does not provide an API for automatic interaction from a third-party application either. Once the equivalence relation was defined, the specification and implementation of the grounding operations were straightforward. All data consumed and produced by this connector were stored in ASCII text files (tab-delimited format).

The third integration scenario was inspired by a study where histologically normal and tumor-associated stromal cells were analysed in order to identify possible changes in the gene expression of prostate cancer cells [55]. In order to cope with a low replication constraint, we needed to use an appropriate statistical method, called *HTself *[56]. However, this method was designed for two-color microarray data, thus a non-trivial data transformation on input data was required. One-color microarray data taken from normal and cancer cells were transformed into (vitual) two-color microarray data and then used as input for the identification of differentiated expressed genes using *HTself*. Then, the obtained data were filtered to be used as input for functional analysis carried out using DAVID.

**Figure 11:**
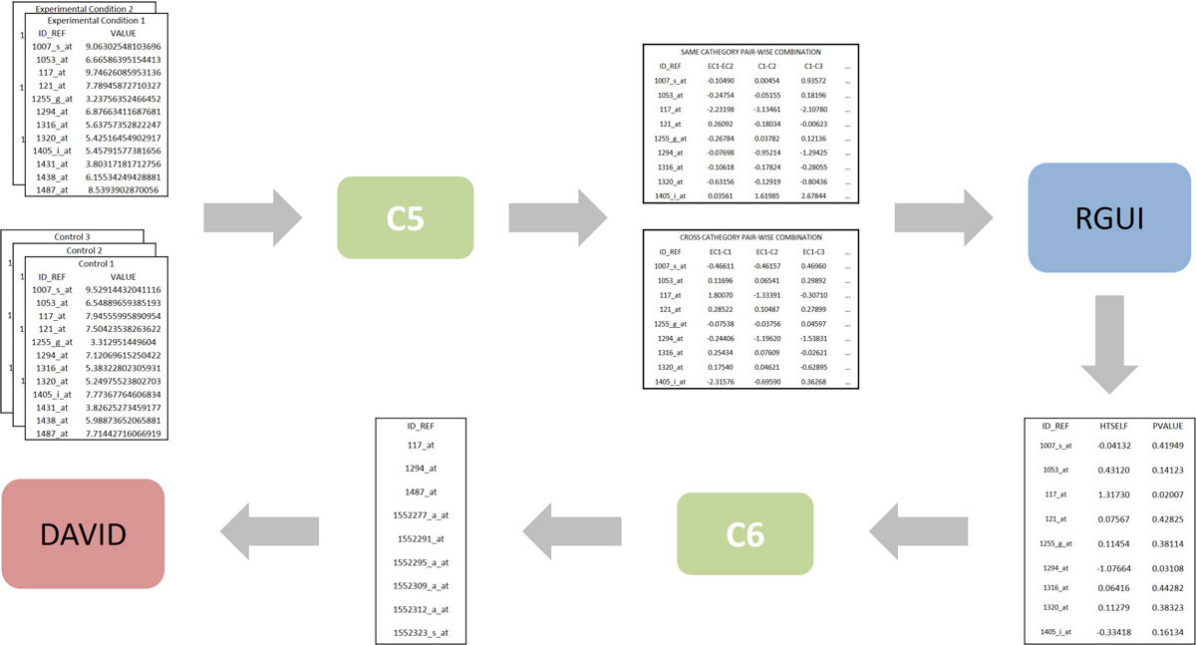
**Integration architecture of one-color microarray data to tools RGUI and DAVID**. A rectangle represents a data source, while a rectangle with rounded corners represents either a tool or a connector. An one-way arrow represents a directed flow of data and/or control.

Figure 11 illustrates the architecture of our third integration scenario with focus on the flow of data. Two connectors were developed to integrate one-color microarray data to RGUI and DAVID. Connector *C5 *transforms one-color microarray data into (virtual) two-color microarray data, so they can be processed by RGUI, while connector *C6 *filters the produced differential gene expression data, so they can be analysed by DAVID.

One-color microarray data was transformed into virtual two-color microarray data by creating sets of ratios using all possible pair-wise comparisons among experiments. This transformation process was carried out in two steps. The first step consisted of creating sets of ratios derived only from samples pertaining to the same cathegory (virtual self-self data), while the second step consisted of creating sets of ratios derived only from actual comparisons among different cathegories.

Connector *C5 *was created to integrate one-color microarray data to RGUI. *C5 *represents a composite connector consisting of two simple connectors: *C5.1 *and *C5.2*, which are responsible for performing steps one and two of the aforementioned transformation process, respectively.

The semantical mapping between concepts representing either consumed or produced data items and concepts from the reference ontology for connector *C5 *was not straightforward either. In two cases, there was no direct association between a concept representing a consumed data item and a concept representing a produced data item. So, equivalence relations had to be created in both cases. In both connectors *C5.1 *and *C5.2*, an equivalence relation was created to associate two instances of the concept *absolute intensity-based value *with one instance of the concept *ratio intensity-based value *according to the aforementioned transformation process (see [55] for a detailed account of this transformation process).

Once the equivalence relations were defined, the specification and implementation of the grounding operations for each simple connector was straightforward. Connectors *C5.1 *and *C5.2 *consumed as input a number of one-color gene expression data files (SOFT format) and produced as output a single virtual two-color gene expression data file each (tab-delimited format). These connectors were then implemented each as a separate Java application, so each connector can be executed and (re)used independently. Connectors *C5.1 *and *C5.2 *were then composed to form connector *C5*, which is responsible for controlling the ordering in which the simple connectors are executed, viz., first *C5.1 *and then *C5.2*. Although connectors *C5.1 *and *C5.2 *can be executed in any order (even concurrently), we have chosen that specific sequencing because, once again, performance is not an issue in the scope of this work.

Similarly to connector *C1*, connector *C5 *as a whole was designed to provide both manual and automatic transfer of control to RGUI (parameter-based selection). The automatic integration of the connector with RGUI was also provided by the RServe API. Since connector *C5 *is capable of providing both forms of transfer of control to RGUI, the automatic integration was implemented independently of connectors *C5.1 *and *C5.2 *in order to maintain a sound implementation structure. Nevertheless, the interaction with RGUI takes place after connector *C5.2 *concludes its execution.

RGUI was used for the identification of differentiated expressed genes on the transformed (virtual) two-color microarray data using *HTself*. RGUI produced as output two numeric values associated to each gene, viz., the resulting value of *HTself *and an associated *p-value*. These data had to be filtered directly by connector *C6 *in order to select for the functional analysis study only those genes whose values for *HTself *and *p-value *are above and below provided thresholds, respectively. As a result, connector *C6 *produced as output a list of genes to be used as input by DAVID.

The semantical mapping between concepts representing either consumed or produced data items and concepts from the reference ontology for connector *C6 *was simpler than for connector *C5*. Initially during the equivalence table construction, two out of three concepts representing a consumed data item (*HTself *and *p-value*) could not be mapped to an equivalent reference ontology concept. In principle, this was not a problem because these concepts were only used as filtering criteria by the connector for the production of the output list of genes. Despite this fact, an equivalence relation was defined to associate instances of the concepts of *gene, HTself *and *p-value *(last two as selection criteria) with instances of the concept *gene*.

Connector *C6 *was also implemented as a separate Java application. This connector provided only manual transfer of control to DAVID, since this tool does not provide an API for automatic interaction from a third-party application either. Once the equivalence relation was defined, the specification and implementation of the grounding operations was straightforward. All data consumed and produced by this connector were stored in ASCII text files (tab-delimited format).

## Discussion

We have developed an ontology-based methodology for the semantic integration of gene expression analysis tools and data sources using software connectors. Our methodology supports not only the access to heterogeneous gene expression data sources but also the definition and implementation of transformation rules on exchanged data. First, we have defined a reference ontology for the gene expression domain. Then, we have defined a number of activities and associated guidelines to prescribe how the development of connectors should be carried out. Finally, we have applied the proposed methodology in the construction of three different integration scenarios involving the use of different tools for the analysis of different types of gene expression data. The availability of a step-by-step methodology based on a reference ontology for the gene expression domain facilitated the development of connectors responsible for the semantic interoperability of the proposed set of data and tools.

The two general approaches used in the semantic integration of bioinformatics tools and databases do not tackle adequately the integration of gene expression analysis tools. In the first approach, ontologies have been used as a common database model to integrate a number of related tools and/or data sets (e.g., Atlas [28], IMGT [31] and IntegromeDB [32]). Although, in principle our reference ontology can be used as basis for the development of a (common) database schema for a number gene expression analysis tools, this is not the main purpose of our reference ontology. GEXPO is used as a reference for mapping concepts representing consumed and produced data items, so they directly or indirectly (through equivalence rules) bear the same semantics as defined in the reference ontology.

In the second approach, mediators have been used to integrate heterogeneous data sources (e.g., TAMBIS [33], SEMEDA [34] and ONTOFUSION [36]). Mediators represent software entities capable of mapping concepts of a global (database) schema to concepts of a local schema. The role played by software connectors in our methodology resembles the role played by mediators, viz., both bridge the gap between global (reference ontology) and local (specific conceptual model). However, mediators are only used to support the translation of queries to local schemas, whereas software connectors can be used not only to perform queries on local databases, but also to relate and transform (sets of) input data onto semantically equivalent output data. Besides, software connectors are primarily intended to integrate analysis tools instead of (multiple) databases in the context of this work.

Semantic integration can also be achieved using a non-systematic approach. In such approach, which could also be based on software connectors, the semantic integration would be achieved on an *ad-hoc *basis, possibly using the concepts of an existing ontology as reference. However, the lack of a systematic methodology for achieving integration would likely result on a more complex, costly and error-prone development process. Furthermore, the lack of structuring guidelines for implementing a connector would possibly reduce the likelihood of reusing existing connectors. Alternatively, integration can also be achieved using a semantic flexibility approach [49]. In this case, no formal mappings are required, i.e., data are exchanged regardless of its meaning. An adapter, associated to each integrated application, receives exchanged data and assigns specific meaning to them according to each specific context of use. Both the non-systematic and the semantic flexibility approaches fall short with respect to our methodology because our systematic approach reduces the chances of misinterpreting data and consequently increases the likelihood of producing meaningful results.

Despite the benefits of our methodology, we must acknowledge a few limitations to our study. In the gene expression domain, there are many different tools and data sources, which can be combined in many different ways. In this sense, the first limitation of our work was the restricted number of integration scenarios in which our methodology was applied. Actually, any methodological work such as ours presents the same limitation. However, our methodology is not restricted to the proposed set of data and tools. On the contrary, it was defined to be as general as possible, so it can be applied in the integration of different tools and data sources in the domain. The proposed integration scenarios were carefully defined. They include the most representative types of gene expression data, as well as tools representing some of the most common gene expression analysis activities. We have also combined tools with different access interfaces and policies. Most of all, we have demonstrated how our methodology was applied to transform actual gene expression data so that semantic integration could be achieved.

The potential limitation of the reference ontology is another source of concern. We were able to successfully map all concepts representing a data item either consumed or produced by the developed set of connectors to a concept defined in the gene expression ontology. Nevertheless, the development of other integration scenarios involving additional sets of data and analysis activities could possibly result in a situation in which such mapping could not be accomplished. In such case, we can fairly assume the reference ontology would be incomplete. Still, since continuous modifications of existing ontologies according to emerging new biological insights represent a common practice in the biomedical domain, our reference ontology could also be subject to a review. Besides, in addition to the set of concepts already defined for the different gene expression measurement approaches described in this paper, we have also included into the reference ontology a set of concepts related to another usual high-throughput gene expression measurement approach, viz., SAGE (see Additional File 1 for the complete gene expression ontology specification).

Finally, despite all structuring guidelines provided by our methodology, there is a lack of (semi-automatic) support for the implementation of the connectors, which can potentially represent a burden for a biologist undertaking this task. Still, the mere existence of systematic methodology containing a set of guidelines for the design and implementation of connectors not only facilitates the development process but also helps reducing potential (conceptual) mistakes that the biologist would more likely incur using an *ad-hoc *development process. In order to facilitate the implementation of connectors, we have developed the GELC API containing a number of classes representing different concepts of our reference ontology. Thus, the biologist can focus on the implementation of the functional blocks of the connector under development. Ultimately, most of the connectors developed in the context of this work were based on nontrivial transformation rules, which are unlikely to be properly generated by any (semi-automatic) code generation tool.

To the best of our knowledge this is the first initiative to provide a systematic methodology for the semantic integration of gene expression analysis tools and data sources using software connectors. Our methodology allows not only the identification of simple equivalence between concepts representing consumed and produced data items but also the definition of (nontrivial) rules in order to establish an equivalence between sets of concepts representing consumed and produced data items. Further, our methodology separates the connector development guidelines from the reference ontology itself. Thus, the same guidelines can be used in the semantic integration of tools and data sources in different (biomedical) domains, such as proteomics, metabolomics and interactomics, provided that a suitable ontology is available to be used as reference for the target domain.

Our ontology-based methodology can be used in the development of semantically integrated analysis environments. The proposed methodolody facilitates the development of connectors capable of achieving semantic interoperability between gene expression analysis tools and data sources. Additionally, developed connectors are capable of supporting both simple and nontrivial processing requirements on exchanged data. Our methodology can be used to create an integrated environment from a set of isolated (non-related) tools and data sources, as well as to extend an existing integrated analysis environment with the integration of new tools and data sources. Thus, our methodology favors the execution of a broader and richer set of analysis activities on available gene expression data.

Furthermore, the set of connectors developed in the context of this work can also be adapted and reused in the integration of other tools and data sources in the domain. For example, connector *C2 *can be adapted to provide integration to other KEGG pathway analysis tools; connectors *C3 *and *C4 *can easily be adapted to process SAGE data; and, finally, connector *C6 *can be adapted to provide integration to other functional and enrichment analysis tools [74]. In this way, connectors can be reused to create other similar integration scenarios.

Semantical integration is pivotal for gene expression analysis. On one hand, a semantically integrated analysis environment is fundamentally important for unveiling new biological knowledge. On the other hand, the lack of semantic integration can, most likely, produce results without biological significance. In many occasions, integration is carried out by someone with insufficient knowledge of the target domain. Even if a domain expert is available, the absence of a systematic approach towards integration favours the arising of (semantical) inconsistencies because integration is based only on tacit knowledge. Our methodology enforces the use of explicit knowledge, since conceptual models must be developed to represent input and output data items. Then, the set of identified concepts are mapped to concepts from a (reference) gene expression ontology, hence contributing for semantic accuracy.

## Conclusions

High-throughput expression measurements of entire transcriptomes can be obtained through different techniques. These data have to be analysed using different tools in order to understand the underlying biological phenomenon. In order to facilitate such analysis, guaranteeing at the same time the soundness of the results, a semantically integrated analysis environment is needed. In this sense, we have developed an ontology-based methodology to support the development of software connectors to integrate gene expression analysis tools and data sources. Our results indicate that the use of our methodology requires the biologist undertaking the integration task to explicitly reason about the underlying semantics of the concepts representing connector input and output data, thus contributing for the correctness and accuracy of the resulting integration as a whole.

Any design methodology can be evaluated according to some general quality properties [75]. Results from the application of our methodology indicate that the proposed methodology adhere to the following properties, viz., *simplicity*, since the methodology uses a minimal set of concepts, which facilitates its learning and application as a whole; *systematicness*, since the methodology provides a stepwise process to guide connector development, in which details are added systematically along the development trajectory; *prescriptiveness*, since the methodology prescribes what should be done rather than what may be done; and, finally, *flexibility*, since the methodology can be used in a variety of situations, without (major) changes or adaptations.

Recently, we have observed an increasing number of gene expression analysis tools becoming available as web services. Web services represent software resources with well-defined interfaces that can be executed remotely through the Internet. Communication and information exchange are carried out using XML-based standard Internet protocols. Similarly to the integration of different tools to create an integrated analysis environment, web services can be integrated to create a composed (analysis) service. Web services whose interfaces are semantically enriched through the use of ontologies are called semantic web services. The availability of semantic descriptions for these services facilitates machine interpretation and, consequently, the automated execution of service compositions. In this sense, future research includes the development and (automatic) composition of semantic web services in the gene expression domain using the reference ontology proposed as part of this work. Additionally, we will also investigate the role played by software connectors to enable proper service compositions.

## Competing interests

The authors declare that they have no competing interests.

## Authors' contributions

FM defined the methodology, helped to define the reference ontology and to implement the integration scenarios. GG defined the reference ontology and helped to draft the manuscript. RV helped to define the reference ontology, defined the integration scenarios and helped to draft the manuscript. CF helped to define the methodology and the reference ontology, implemented the integration scenarios and drafted the manuscript. All authors read and approved the final manuscript.

## Supplementary Material

Additional file 1 **OWL Specification of the Gene Expression Ontology**. Complete specification of the Gene Expression Ontology in OWL. Click here for file

Additional File 2 **Connectors C1 and C2 Implementation**. Connectors *C1 *and *C2 *source code and documentation (javadoc format). Click here for file

Additional File 3 **GELC API**. GELC API binary code (jar format) and documentation (javadoc format). Click here for file

## Declarations

Publication for this article has been funded by the Brazilian Ministry of Education (CAPES).

This article has been published as part of *BMC Genomics *Volume 14 Supplement 6, 2013: Proceedings of the International Conference of the Brazilian Association for Bioinformatics and Computational Biology (X-meeting 2012). The full contents of the supplement are available online at http://www.biomedcentral.com/bmcgenomics/supplements/14/S6.
