# Supplementary material for: Semantic integration of gene expression analysis tools and data sources using software connectors
Source: BMC Genomics. 2013 Oct 25;14(Suppl 6):S2. doi: 10.1186/1471-2164-14-S6-S2 (PMC3908368; doi:10.1186/1471-2164-14-S6-S2)
Supplement: Additional File 2 — Connectors C1 and C2 Implementation. Connectors C1 and C2 source code and documentation (javadoc format). [file 1471-2164-14-S6-S2-S2.zip › connector_c1/documentation/c1/C1.html]

C1


---


|  |  |  |  |  |  |  |  |  |  |
| --- | --- | --- | --- | --- | --- | --- | --- | --- | --- |
| |  |  |  |  |  |  |  | | --- | --- | --- | --- | --- | --- | --- | | **Package** | **Class** | **Use** | **Tree** | **Deprecated** | **Index** | **Help** | | |  |
| PREV CLASS   NEXT CLASS | **FRAMES**    **NO FRAMES**     **All Classes** |
| SUMMARY: NESTED | FIELD | CONSTR | METHOD | DETAIL: FIELD | CONSTR | METHOD |


---


## c1 Class C1

```
java.lang.Object
  c1.C1
```

---

``` public class C1 extends java.lang.Object ```

This class implements connector C1.

---

| **Constructor Summary** | |
| --- | --- |
| `C1()` |


| **Method Summary** | |
| --- | --- |
| `static void` | `dataInputProcessing(java.lang.String inputDirectory, java.util.List<java.util.List<java.lang.String>> inputStringList)`             Do data input processing. |
| `static void` | `dataOutputProcessing(java.lang.String outputDirectory, java.lang.String outputFile, java.util.List<java.lang.String> outputList)`             Do data output processing. |
| `static void` | `integrateWithRServer(java.lang.String serverAddr, java.lang.String rInteractionDirectory, java.lang.String rInputFile, java.lang.String rOutputFile)`             Do integration with Rserve. |
| `static void` | `lifting(java.util.List<java.util.List<java.lang.String>> inputStringList, java.util.List<gelc.Gene> outputGeneList, java.util.List<gelc.ExperimentalCondition> outputConditionList, java.util.List<java.util.List<gelc.RatioIntensityBasedValue>> ratioExpressionValueLists)`             Do lifting. |
| `static void` | `lowering(java.util.List<gelc.Gene> geneList, java.util.List<gelc.ExperimentalCondition> conditionList, java.util.List<java.util.List<gelc.RatioIntensityBasedValue>> ratioExpressionValueLists, java.util.List<java.lang.String> outputList)`             Do lowering. |
| `static void` | `main(java.lang.String[] args)` |

| **Methods inherited from class java.lang.Object** |
| --- |
| `clone, equals, finalize, getClass, hashCode, notify, notifyAll, toString, wait, wait, wait` |

| **Constructor Detail** |
| --- |

### C1

```
public C1()
```


| **Method Detail** |
| --- |

### main

```
public static void main(java.lang.String[] args)
```

:   **Parameters:**: `args` - the command line arguments (args[0] = input directory; args[1] = output directory; args[2] = output file; args[3] = r server internet address; args[4] = r output file)

---


### dataInputProcessing

```
public static void dataInputProcessing(java.lang.String inputDirectory,
                                       java.util.List<java.util.List<java.lang.String>> inputStringList)
```

:   Do data input processing.

    :   **Parameters:**: `inputDirectory` - the input directory identifier: `inputStringList` - the connector input lists of string elements

---


### lifting

```
public static void lifting(java.util.List<java.util.List<java.lang.String>> inputStringList,
                           java.util.List<gelc.Gene> outputGeneList,
                           java.util.List<gelc.ExperimentalCondition> outputConditionList,
                           java.util.List<java.util.List<gelc.RatioIntensityBasedValue>> ratioExpressionValueLists)
```

:   Do lifting.

    :   **Parameters:**: `inputStringList` - the input lists of string elements to be lifted: `outputGeneList` - the lifted list of genes: `outputConditionList` - the lifted list of experimental conditions: `ratioExpressionValueLists` - the lifted lists of ratio intensity-based gene expression values

---


### lowering

```
public static void lowering(java.util.List<gelc.Gene> geneList,
                            java.util.List<gelc.ExperimentalCondition> conditionList,
                            java.util.List<java.util.List<gelc.RatioIntensityBasedValue>> ratioExpressionValueLists,
                            java.util.List<java.lang.String> outputList)
```

:   Do lowering.

    :   **Parameters:**: `geneList` - the connector lifted list of genes: `conditionList` - the connector lifted list of experimental conditions: `ratioExpressionValueLists` - the connector lifted lists of ratio intensity-based gene expression values: `outputList` - the lowered list of string elements

---


### dataOutputProcessing

```
public static void dataOutputProcessing(java.lang.String outputDirectory,
                                        java.lang.String outputFile,
                                        java.util.List<java.lang.String> outputList)
```

:   Do data output processing.

    :   **Parameters:**: `outputDirectory` - the output directory identifier: `outputFile` - the output file identifier: `outputList` - the connector produced list of string elements

---


### integrateWithRServer

```
public static void integrateWithRServer(java.lang.String serverAddr,
                                        java.lang.String rInteractionDirectory,
                                        java.lang.String rInputFile,
                                        java.lang.String rOutputFile)
```

:   Do integration with Rserve.

    :   **Parameters:**: `serverAddr` - the Rserve (IP) addresss: `rInteractionDirectory` - the output directory containing the files sent/received to/from Rserve: `rInputFile` - the input file to be sent to Rserve: `rOutputFile` - the output file received from Rserve


---


|  |  |  |  |  |  |  |  |  |  |
| --- | --- | --- | --- | --- | --- | --- | --- | --- | --- |
| |  |  |  |  |  |  |  | | --- | --- | --- | --- | --- | --- | --- | | **Package** | **Class** | **Use** | **Tree** | **Deprecated** | **Index** | **Help** | | |  |
| PREV CLASS   NEXT CLASS | **FRAMES**    **NO FRAMES**     **All Classes** |
| SUMMARY: NESTED | FIELD | CONSTR | METHOD | DETAIL: FIELD | CONSTR | METHOD |


---
