# Supplementary material for: Semantic integration of gene expression analysis tools and data sources using software connectors
Source: BMC Genomics. 2013 Oct 25;14(Suppl 6):S2. doi: 10.1186/1471-2164-14-S6-S2 (PMC3908368; doi:10.1186/1471-2164-14-S6-S2)
Supplement: Additional File 2 — Connectors C1 and C2 Implementation. Connectors C1 and C2 source code and documentation (javadoc format). [file 1471-2164-14-S6-S2-S2.zip › connector_c2/documentation/c2/GeneMap.html]

GeneMap


---


|  |  |  |  |  |  |  |  |  |  |
| --- | --- | --- | --- | --- | --- | --- | --- | --- | --- |
| |  |  |  |  |  |  |  | | --- | --- | --- | --- | --- | --- | --- | | **Package** | **Class** | **Use** | **Tree** | **Deprecated** | **Index** | **Help** | | |  |
| **PREV CLASS**   **NEXT CLASS** | **FRAMES**    **NO FRAMES**     **All Classes** |
| SUMMARY: NESTED | FIELD | CONSTR | METHOD | DETAIL: FIELD | CONSTR | METHOD |


---


## c2 Class GeneMap

```
java.lang.Object
  c2.GeneMap
```

---

``` public class GeneMap extends java.lang.Object ```

This class maps experiment specific gene identifiers to KEGG identifiers.

---

| **Constructor Summary** | |
| --- | --- |
| `GeneMap(java.util.List<KEGGIdentifier> info)`             Constructor GeneMap. |


| **Method Summary** | |
| --- | --- |
| `java.lang.String` | `getKeggIdentifier(int geneId)`             Obtains the KEGG identifier. |

| **Methods inherited from class java.lang.Object** |
| --- |
| `clone, equals, finalize, getClass, hashCode, notify, notifyAll, toString, wait, wait, wait` |

| **Constructor Detail** |
| --- |

### GeneMap

```
public GeneMap(java.util.List<KEGGIdentifier> info)
```

:   Constructor GeneMap.

    **Parameters:**: `info` - the list of KEGG identifiers


| **Method Detail** |
| --- |

### getKeggIdentifier

```
public java.lang.String getKeggIdentifier(int geneId)
```

:   Obtains the KEGG identifier.

    :   **Parameters:**: `geneId` - the experiment-specific gene identifier **Returns:**: the corresponding KEGG identifier


---


|  |  |  |  |  |  |  |  |  |  |
| --- | --- | --- | --- | --- | --- | --- | --- | --- | --- |
| |  |  |  |  |  |  |  | | --- | --- | --- | --- | --- | --- | --- | | **Package** | **Class** | **Use** | **Tree** | **Deprecated** | **Index** | **Help** | | |  |
| **PREV CLASS**   **NEXT CLASS** | **FRAMES**    **NO FRAMES**     **All Classes** |
| SUMMARY: NESTED | FIELD | CONSTR | METHOD | DETAIL: FIELD | CONSTR | METHOD |


---
