# Supplementary material for: Semantic integration of gene expression analysis tools and data sources using software connectors
Source: BMC Genomics. 2013 Oct 25;14(Suppl 6):S2. doi: 10.1186/1471-2164-14-S6-S2 (PMC3908368; doi:10.1186/1471-2164-14-S6-S2)
Supplement: Additional File 2 — Connectors C1 and C2 Implementation. Connectors C1 and C2 source code and documentation (javadoc format). [file 1471-2164-14-S6-S2-S2.zip › connector_c2/documentation/c2/GeneMapParser.html]

GeneMapParser


---


|  |  |  |  |  |  |  |  |  |  |
| --- | --- | --- | --- | --- | --- | --- | --- | --- | --- |
| |  |  |  |  |  |  |  | | --- | --- | --- | --- | --- | --- | --- | | **Package** | **Class** | **Use** | **Tree** | **Deprecated** | **Index** | **Help** | | |  |
| **PREV CLASS**   **NEXT CLASS** | **FRAMES**    **NO FRAMES**     **All Classes** |
| SUMMARY: NESTED | FIELD | CONSTR | METHOD | DETAIL: FIELD | CONSTR | METHOD |


---


## c2 Class GeneMapParser

```
java.lang.Object
  c2.GeneMapParser
```

---

``` public class GeneMapParser extends java.lang.Object ```

This class parses a file containing experiment specific gene identifiers and their
correspoing KEGG identifiers and returns a GeneMap object.

---

| **Constructor Summary** | |
| --- | --- |
| `GeneMapParser()` |


| **Method Summary** | |
| --- | --- |
| `static GeneMap` | `parseGeneMapFileDescription(java.lang.String inputDirectory, java.lang.String inputFile)`             Parses a file containing experiment specific gene identifiers and their correspoing KEGG identifiers and returns a GeneMap object. |

| **Methods inherited from class java.lang.Object** |
| --- |
| `clone, equals, finalize, getClass, hashCode, notify, notifyAll, toString, wait, wait, wait` |

| **Constructor Detail** |
| --- |

### GeneMapParser

```
public GeneMapParser()
```


| **Method Detail** |
| --- |

### parseGeneMapFileDescription

```
public static GeneMap parseGeneMapFileDescription(java.lang.String inputDirectory,
                                                  java.lang.String inputFile)
```

:   Parses a file containing experiment specific gene identifiers and their
    correspoing KEGG identifiers and returns a GeneMap object.

    :   **Parameters:**: `inputDirectory` - the experiment-specific gene identifier to KEGG identifier mapping directory identifier: `inputFile` - the input experiment-specific gene identifier to KEGG identifier mapping file identifier **Returns:**: the corresponding GeneMap object


---


|  |  |  |  |  |  |  |  |  |  |
| --- | --- | --- | --- | --- | --- | --- | --- | --- | --- |
| |  |  |  |  |  |  |  | | --- | --- | --- | --- | --- | --- | --- | | **Package** | **Class** | **Use** | **Tree** | **Deprecated** | **Index** | **Help** | | |  |
| **PREV CLASS**   **NEXT CLASS** | **FRAMES**    **NO FRAMES**     **All Classes** |
| SUMMARY: NESTED | FIELD | CONSTR | METHOD | DETAIL: FIELD | CONSTR | METHOD |


---
