# Supplementary material for: Semantic integration of gene expression analysis tools and data sources using software connectors
Source: BMC Genomics. 2013 Oct 25;14(Suppl 6):S2. doi: 10.1186/1471-2164-14-S6-S2 (PMC3908368; doi:10.1186/1471-2164-14-S6-S2)
Supplement: Additional File 2 — Connectors C1 and C2 Implementation. Connectors C1 and C2 source code and documentation (javadoc format). [file 1471-2164-14-S6-S2-S2.zip › connector_c2/documentation/c2/package-frame.html]

c2


c2

|  |
| --- |
| Classes    C2   GeneMap   GeneMapParser   KEGGIdentifier |
