# Supplementary material for: Semantic integration of gene expression analysis tools and data sources using software connectors
Source: BMC Genomics. 2013 Oct 25;14(Suppl 6):S2. doi: 10.1186/1471-2164-14-S6-S2 (PMC3908368; doi:10.1186/1471-2164-14-S6-S2)
Supplement: Additional File 2 — Connectors C1 and C2 Implementation. Connectors C1 and C2 source code and documentation (javadoc format). [file 1471-2164-14-S6-S2-S2.zip › connector_c2/documentation/c2/package-summary.html]

c2


---


|  |  |  |  |  |  |  |  |  |  |
| --- | --- | --- | --- | --- | --- | --- | --- | --- | --- |
| |  |  |  |  |  |  |  | | --- | --- | --- | --- | --- | --- | --- | | **Package** | Class | **Use** | **Tree** | **Deprecated** | **Index** | **Help** | | |  |
| PREV PACKAGE   NEXT PACKAGE | **FRAMES**    **NO FRAMES**     **All Classes** |


---

## Package c2

| **Class Summary** | |
| --- | --- |
| **C2** | This class implements connector C2. |
| **GeneMap** | This class maps experiment specific gene identifiers to KEGG identifiers. |
| **GeneMapParser** | This class parses a file containing experiment specific gene identifiers and their correspoing KEGG identifiers and returns a GeneMap object. |
| **KEGGIdentifier** | This class represents a KEGG identifier. |

---


|  |  |  |  |  |  |  |  |  |  |
| --- | --- | --- | --- | --- | --- | --- | --- | --- | --- |
| |  |  |  |  |  |  |  | | --- | --- | --- | --- | --- | --- | --- | | **Package** | Class | **Use** | **Tree** | **Deprecated** | **Index** | **Help** | | |  |
| PREV PACKAGE   NEXT PACKAGE | **FRAMES**    **NO FRAMES**     **All Classes** |


---
