# Supplementary material for: Semantic integration of gene expression analysis tools and data sources using software connectors
Source: BMC Genomics. 2013 Oct 25;14(Suppl 6):S2. doi: 10.1186/1471-2164-14-S6-S2 (PMC3908368; doi:10.1186/1471-2164-14-S6-S2)
Supplement: Additional File 2 — Connectors C1 and C2 Implementation. Connectors C1 and C2 source code and documentation (javadoc format). [file 1471-2164-14-S6-S2-S2.zip › connector_c2/documentation/index-files/index-3.html]

G-Index


---


|  |  |  |  |  |  |  |  |  |  |
| --- | --- | --- | --- | --- | --- | --- | --- | --- | --- |
| |  |  |  |  |  |  |  | | --- | --- | --- | --- | --- | --- | --- | | **Package** | Class | Use | **Tree** | **Deprecated** | **Index** | **Help** | | |  |
| **PREV LETTER**   **NEXT LETTER** | **FRAMES**    **NO FRAMES**     **All Classes** |


C D G K L M P 

---


## **G**

**GeneMap** - Class in c2: This class maps experiment specific gene identifiers to KEGG identifiers. **GeneMap(List<KEGGIdentifier>)** - Constructor for class c2.GeneMap: Constructor GeneMap. **GeneMapParser** - Class in c2: This class parses a file containing experiment specific gene identifiers and their correspoing KEGG identifiers and returns a GeneMap object. **GeneMapParser()** - Constructor for class c2.GeneMapParser: **getIdentifier()** - Method in class c2.KEGGIdentifier: Obtains the KEGG identifier. **getKeggIdentifier(int)** - Method in class c2.GeneMap: Obtains the KEGG identifier.

---


|  |  |  |  |  |  |  |  |  |  |
| --- | --- | --- | --- | --- | --- | --- | --- | --- | --- |
| |  |  |  |  |  |  |  | | --- | --- | --- | --- | --- | --- | --- | | **Package** | Class | Use | **Tree** | **Deprecated** | **Index** | **Help** | | |  |
| **PREV LETTER**   **NEXT LETTER** | **FRAMES**    **NO FRAMES**     **All Classes** |


C D G K L M P 

---
