# Supplementary material for: Semantic integration of gene expression analysis tools and data sources using software connectors
Source: BMC Genomics. 2013 Oct 25;14(Suppl 6):S2. doi: 10.1186/1471-2164-14-S6-S2 (PMC3908368; doi:10.1186/1471-2164-14-S6-S2)
Supplement: Additional File 2 — Connectors C1 and C2 Implementation. Connectors C1 and C2 source code and documentation (javadoc format). [file 1471-2164-14-S6-S2-S2.zip › connector_c2/documentation/index-files/index-5.html]

L-Index


---


|  |  |  |  |  |  |  |  |  |  |
| --- | --- | --- | --- | --- | --- | --- | --- | --- | --- |
| |  |  |  |  |  |  |  | | --- | --- | --- | --- | --- | --- | --- | | **Package** | Class | Use | **Tree** | **Deprecated** | **Index** | **Help** | | |  |
| **PREV LETTER**   **NEXT LETTER** | **FRAMES**    **NO FRAMES**     **All Classes** |


C D G K L M P 

---


## **L**

**lifting(String, String, List<String>, String, double, List<Gene>, List<RatioIntensityBasedValue<GeneRegulation>>)** - Static method in class c2.C2: Do lifting. **lowering(List<Gene>, List<RatioIntensityBasedValue<GeneRegulation>>, List<String>)** - Static method in class c2.C2: Do lowering.

---


|  |  |  |  |  |  |  |  |  |  |
| --- | --- | --- | --- | --- | --- | --- | --- | --- | --- |
| |  |  |  |  |  |  |  | | --- | --- | --- | --- | --- | --- | --- | | **Package** | Class | Use | **Tree** | **Deprecated** | **Index** | **Help** | | |  |
| **PREV LETTER**   **NEXT LETTER** | **FRAMES**    **NO FRAMES**     **All Classes** |


C D G K L M P 

---
