# Supplementary material for: Semantic integration of gene expression analysis tools and data sources using software connectors
Source: BMC Genomics. 2013 Oct 25;14(Suppl 6):S2. doi: 10.1186/1471-2164-14-S6-S2 (PMC3908368; doi:10.1186/1471-2164-14-S6-S2)
Supplement: Additional File 2 — Connectors C1 and C2 Implementation. Connectors C1 and C2 source code and documentation (javadoc format). [file 1471-2164-14-S6-S2-S2.zip › connector_c2/documentation/index.html]

Generated Documentation (Untitled)


<H2>
Frame Alert</H2>
<P>
This document is designed to be viewed using the frames feature. If you see this message, you are using a non-frame-capable web client.
<BR>
Link to<A HREF="c2/package-summary.html">Non-frame version.</A>
