# Supplementary material for: Semantic integration of gene expression analysis tools and data sources using software connectors
Source: BMC Genomics. 2013 Oct 25;14(Suppl 6):S2. doi: 10.1186/1471-2164-14-S6-S2 (PMC3908368; doi:10.1186/1471-2164-14-S6-S2)
Supplement: Additional File 3 — GELC API. GELC API binary code (jar format) and documentation (javadoc format). [file 1471-2164-14-S6-S2-S3.zip › documentation/gelc/AbsoluteIntensityBasedValue.html]

AbsoluteIntensityBasedValue (GELC API)


---


|  |  |  |  |  |  |  |  |  |  |
| --- | --- | --- | --- | --- | --- | --- | --- | --- | --- |
| |  |  |  |  |  |  |  | | --- | --- | --- | --- | --- | --- | --- | | **Package** | **Class** | **Use** | **Tree** | **Deprecated** | **Index** | **Help** | | | *Gene Expression Library Class API v1.0* |
| **PREV CLASS**   **NEXT CLASS** | **FRAMES**    **NO FRAMES**     **All Classes** |
| SUMMARY: NESTED | FIELD | CONSTR | METHOD | DETAIL: FIELD | CONSTR | METHOD |


---


## gelc Class AbsoluteIntensityBasedValue

```
java.lang.Object
  gelc.AbsoluteIntensityBasedValue
```

---

``` public class AbsoluteIntensityBasedValue extends java.lang.Object ```

This class represents an absolute intensity-based gene expression value.

**Author:**
:   C. R. G. de Farias

---

| **Constructor Summary** | |
| --- | --- |
| `AbsoluteIntensityBasedValue(double value)`             Constructor AbsoluteIntensityBasedValue. |


| **Method Summary** | |
| --- | --- |
| `boolean` | `equals(AbsoluteIntensityBasedValue value)`             Compares this object against the specified object. |
| `double` | `getValue()`             Obtains the absolute intensity-based value itself. |
| `java.lang.String` | `toString()`             Returns a string representation of this AbsoluteIntensityBasedValue object. |

| **Methods inherited from class java.lang.Object** |
| --- |
| `clone, equals, finalize, getClass, hashCode, notify, notifyAll, wait, wait, wait` |

| **Constructor Detail** |
| --- |

### AbsoluteIntensityBasedValue

```
public AbsoluteIntensityBasedValue(double value)
```

:   Constructor AbsoluteIntensityBasedValue.

    **Parameters:**: `value` - the value itself


| **Method Detail** |
| --- |

### getValue

```
public double getValue()
```

:   Obtains the absolute intensity-based value itself.

    :   **Returns:**: value itself

---


### equals

```
public boolean equals(AbsoluteIntensityBasedValue value)
```

:   Compares this object against the specified object.

    :   **Parameters:**: `value` - the object to compare with **Returns:**: true if the objects are the same; false otherwise

---


### toString

```
public java.lang.String toString()
```

:   Returns a string representation of this AbsoluteIntensityBasedValue object.

    :   **Overrides:**: `toString` in class `java.lang.Object`
    :   **Returns:**: a string representation of this object


---


|  |  |  |  |  |  |  |  |  |  |
| --- | --- | --- | --- | --- | --- | --- | --- | --- | --- |
| |  |  |  |  |  |  |  | | --- | --- | --- | --- | --- | --- | --- | | **Package** | **Class** | **Use** | **Tree** | **Deprecated** | **Index** | **Help** | | | *Gene Expression Library Class API v1.0* |
| **PREV CLASS**   **NEXT CLASS** | **FRAMES**    **NO FRAMES**     **All Classes** |
| SUMMARY: NESTED | FIELD | CONSTR | METHOD | DETAIL: FIELD | CONSTR | METHOD |


---
