# Supplementary material for: Semantic integration of gene expression analysis tools and data sources using software connectors
Source: BMC Genomics. 2013 Oct 25;14(Suppl 6):S2. doi: 10.1186/1471-2164-14-S6-S2 (PMC3908368; doi:10.1186/1471-2164-14-S6-S2)
Supplement: Additional File 3 — GELC API. GELC API binary code (jar format) and documentation (javadoc format). [file 1471-2164-14-S6-S2-S3.zip › documentation/gelc/class-use/GeneRegulation.html]

Uses of Class gelc.GeneRegulation (GELC API)


---


|  |  |  |  |  |  |  |  |  |  |
| --- | --- | --- | --- | --- | --- | --- | --- | --- | --- |
| |  |  |  |  |  |  |  | | --- | --- | --- | --- | --- | --- | --- | | **Package** | **Class** | **Use** | **Tree** | **Deprecated** | **Index** | **Help** | | | *Gene Expression Library Class API v1.0* |
| PREV   NEXT | **FRAMES**    **NO FRAMES**     **All Classes** |


---


## **Uses of Class gelc.GeneRegulation**


| Uses of GeneRegulation in gelc | |
| --- | --- |

| Methods in gelc that return GeneRegulation | |
| --- | --- |
| `static GeneRegulation` | `GeneRegulation.valueOf(java.lang.String name)`             Returns the enum constant of this type with the specified name. |
| `static GeneRegulation[]` | `GeneRegulation.values()`             Returns an array containing the constants of this enum type, in the order they are declared. |

---


|  |  |  |  |  |  |  |  |  |  |
| --- | --- | --- | --- | --- | --- | --- | --- | --- | --- |
| |  |  |  |  |  |  |  | | --- | --- | --- | --- | --- | --- | --- | | **Package** | **Class** | **Use** | **Tree** | **Deprecated** | **Index** | **Help** | | | *Gene Expression Library Class API v1.0* |
| PREV   NEXT | **FRAMES**    **NO FRAMES**     **All Classes** |


---
