# Supplementary material for: Semantic integration of gene expression analysis tools and data sources using software connectors
Source: BMC Genomics. 2013 Oct 25;14(Suppl 6):S2. doi: 10.1186/1471-2164-14-S6-S2 (PMC3908368; doi:10.1186/1471-2164-14-S6-S2)
Supplement: Additional File 3 — GELC API. GELC API binary code (jar format) and documentation (javadoc format). [file 1471-2164-14-S6-S2-S3.zip › documentation/gelc/GeneRegulation.html]

GeneRegulation (GELC API)


---


|  |  |  |  |  |  |  |  |  |  |
| --- | --- | --- | --- | --- | --- | --- | --- | --- | --- |
| |  |  |  |  |  |  |  | | --- | --- | --- | --- | --- | --- | --- | | **Package** | **Class** | **Use** | **Tree** | **Deprecated** | **Index** | **Help** | | | *Gene Expression Library Class API v1.0* |
| **PREV CLASS**   **NEXT CLASS** | **FRAMES**    **NO FRAMES**     **All Classes** |
| SUMMARY: NESTED | ENUM CONSTANTS | FIELD | METHOD | DETAIL: ENUM CONSTANTS | FIELD | METHOD |


---


## gelc Enum GeneRegulation

```
java.lang.Object
  java.lang.Enum<GeneRegulation>
      gelc.GeneRegulation
```

**All Implemented Interfaces:**: java.io.Serializable, java.lang.Comparable<GeneRegulation>

---

``` public enum GeneRegulation extends java.lang.Enum<GeneRegulation> ```

This class represents a specific representation of a ratio intensity-based gene expression value.

**Author:**
:   C. R. G. de Farias

---

| **Enum Constant Summary** | |
| --- | --- |
| `downregulation` |
| `noregulation` |
| `undefined` |
| `upregulation` |


| **Method Summary** | |
| --- | --- |
| `static GeneRegulation` | `valueOf(java.lang.String name)`             Returns the enum constant of this type with the specified name. |
| `static GeneRegulation[]` | `values()`             Returns an array containing the constants of this enum type, in the order they are declared. |

| **Methods inherited from class java.lang.Enum** |
| --- |
| `clone, compareTo, equals, finalize, getDeclaringClass, hashCode, name, ordinal, toString, valueOf` |

| **Methods inherited from class java.lang.Object** |
| --- |
| `getClass, notify, notifyAll, wait, wait, wait` |

| **Enum Constant Detail** |
| --- |

### upregulation

```
public static final GeneRegulation upregulation
```

---


### downregulation

```
public static final GeneRegulation downregulation
```

---


### noregulation

```
public static final GeneRegulation noregulation
```

---


### undefined

```
public static final GeneRegulation undefined
```


| **Method Detail** |
| --- |

### values

```
public static GeneRegulation[] values()
```

:   Returns an array containing the constants of this enum type, in
    the order they are declared. This method may be used to iterate
    over the constants as follows:

    ```
    for (GeneRegulation c : GeneRegulation.values())
        System.out.println(c);
    ```

    :   **Returns:**: an array containing the constants of this enum type, in the order they are declared

---


### valueOf

```
public static GeneRegulation valueOf(java.lang.String name)
```

:   Returns the enum constant of this type with the specified name.
    The string must match *exactly* an identifier used to declare an
    enum constant in this type. (Extraneous whitespace characters are
    not permitted.)

    :   **Parameters:**: `name` - the name of the enum constant to be returned. **Returns:**: the enum constant with the specified name **Throws:**: `java.lang.IllegalArgumentException` - if this enum type has no constant with the specified name: `java.lang.NullPointerException` - if the argument is null


---


|  |  |  |  |  |  |  |  |  |  |
| --- | --- | --- | --- | --- | --- | --- | --- | --- | --- |
| |  |  |  |  |  |  |  | | --- | --- | --- | --- | --- | --- | --- | | **Package** | **Class** | **Use** | **Tree** | **Deprecated** | **Index** | **Help** | | | *Gene Expression Library Class API v1.0* |
| **PREV CLASS**   **NEXT CLASS** | **FRAMES**    **NO FRAMES**     **All Classes** |
| SUMMARY: NESTED | ENUM CONSTANTS | FIELD | METHOD | DETAIL: ENUM CONSTANTS | FIELD | METHOD |


---
