# Supplementary material for: Semantic integration of gene expression analysis tools and data sources using software connectors
Source: BMC Genomics. 2013 Oct 25;14(Suppl 6):S2. doi: 10.1186/1471-2164-14-S6-S2 (PMC3908368; doi:10.1186/1471-2164-14-S6-S2)
Supplement: Additional File 3 — GELC API. GELC API binary code (jar format) and documentation (javadoc format). [file 1471-2164-14-S6-S2-S3.zip › documentation/gelc/package-frame.html]

gelc (GELC API)


gelc

|  |
| --- |
| Classes    AbsoluteCDNAReadsCountingBasedValue   AbsoluteIntensityBasedValue   AbsoluteSAGETagsCountingBasedValue   CDNARead   ExperimentalCondition   Gene   MatureTranscript   RatioIntensityBasedValue   RelativeCDNAReadsCountingBasedValue   RelativeSAGETagsCountingBasedValue   SAGETag |

|  |
| --- |
| Enums    GeneRegulation |
