# Supplementary material for: Semantic integration of gene expression analysis tools and data sources using software connectors
Source: BMC Genomics. 2013 Oct 25;14(Suppl 6):S2. doi: 10.1186/1471-2164-14-S6-S2 (PMC3908368; doi:10.1186/1471-2164-14-S6-S2)
Supplement: Additional File 3 — GELC API. GELC API binary code (jar format) and documentation (javadoc format). [file 1471-2164-14-S6-S2-S3.zip › documentation/gelc/package-summary.html]

gelc (GELC API)


---


|  |  |  |  |  |  |  |  |  |  |
| --- | --- | --- | --- | --- | --- | --- | --- | --- | --- |
| |  |  |  |  |  |  |  | | --- | --- | --- | --- | --- | --- | --- | | **Package** | Class | **Use** | **Tree** | **Deprecated** | **Index** | **Help** | | | *Gene Expression Library Class API v1.0* |
| PREV PACKAGE   NEXT PACKAGE | **FRAMES**    **NO FRAMES**     **All Classes** |


---

## Package gelc

| **Class Summary** | |
| --- | --- |
| **AbsoluteCDNAReadsCountingBasedValue** | This class represents an absolute cDNA reads counting-based gene expression value. |
| **AbsoluteIntensityBasedValue** | This class represents an absolute intensity-based gene expression value. |
| **AbsoluteSAGETagsCountingBasedValue** | This class represents an absolute SAGE tags counting-based gene expression value. |
| **CDNARead<E>** | This class represents a cDNA read. |
| **ExperimentalCondition** | This class represents an experimental condition. |
| **Gene** | This class represents a gene. |
| **MatureTranscript** | This class represents a mature transcript. |
| **RatioIntensityBasedValue<E>** | This class represents a ratio intensity-based gene expression value. |
| **RelativeCDNAReadsCountingBasedValue<E>** | This class represents a relative cDNA reads counting-based gene expression value. |
| **RelativeSAGETagsCountingBasedValue<E>** | This class represents a relative SAGE tags counting-based gene expression value. |
| **SAGETag** | This class represents a SAGE tag. |

| **Enum Summary** | |
| --- | --- |
| **GeneRegulation** | This class represents a specific representation of a ratio intensity-based gene expression value. |

---


|  |  |  |  |  |  |  |  |  |  |
| --- | --- | --- | --- | --- | --- | --- | --- | --- | --- |
| |  |  |  |  |  |  |  | | --- | --- | --- | --- | --- | --- | --- | | **Package** | Class | **Use** | **Tree** | **Deprecated** | **Index** | **Help** | | | *Gene Expression Library Class API v1.0* |
| PREV PACKAGE   NEXT PACKAGE | **FRAMES**    **NO FRAMES**     **All Classes** |


---
