# Supplementary material for: Semantic integration of gene expression analysis tools and data sources using software connectors
Source: BMC Genomics. 2013 Oct 25;14(Suppl 6):S2. doi: 10.1186/1471-2164-14-S6-S2 (PMC3908368; doi:10.1186/1471-2164-14-S6-S2)
Supplement: Additional File 3 — GELC API. GELC API binary code (jar format) and documentation (javadoc format). [file 1471-2164-14-S6-S2-S3.zip › documentation/gelc/package-use.html]

Uses of Package gelc (GELC API)


---


|  |  |  |  |  |  |  |  |  |  |
| --- | --- | --- | --- | --- | --- | --- | --- | --- | --- |
| |  |  |  |  |  |  |  | | --- | --- | --- | --- | --- | --- | --- | | **Package** | Class | **Use** | **Tree** | **Deprecated** | **Index** | **Help** | | | *Gene Expression Library Class API v1.0* |
| PREV   NEXT | **FRAMES**    **NO FRAMES**     **All Classes** |


---


## **Uses of Package gelc**


| Classes in gelc used by gelc | |
| --- | --- |
| ****AbsoluteCDNAReadsCountingBasedValue****             This class represents an absolute cDNA reads counting-based gene expression value. |
| ****AbsoluteIntensityBasedValue****             This class represents an absolute intensity-based gene expression value. |
| ****AbsoluteSAGETagsCountingBasedValue****             This class represents an absolute SAGE tags counting-based gene expression value. |
| ****CDNARead****             This class represents a cDNA read. |
| ****ExperimentalCondition****             This class represents an experimental condition. |
| ****Gene****             This class represents a gene. |
| ****GeneRegulation****             This class represents a specific representation of a ratio intensity-based gene expression value. |
| ****MatureTranscript****             This class represents a mature transcript. |
| ****RatioIntensityBasedValue****             This class represents a ratio intensity-based gene expression value. |
| ****RelativeCDNAReadsCountingBasedValue****             This class represents a relative cDNA reads counting-based gene expression value. |
| ****RelativeSAGETagsCountingBasedValue****             This class represents a relative SAGE tags counting-based gene expression value. |
| ****SAGETag****             This class represents a SAGE tag. |

---


|  |  |  |  |  |  |  |  |  |  |
| --- | --- | --- | --- | --- | --- | --- | --- | --- | --- |
| |  |  |  |  |  |  |  | | --- | --- | --- | --- | --- | --- | --- | | **Package** | Class | **Use** | **Tree** | **Deprecated** | **Index** | **Help** | | | *Gene Expression Library Class API v1.0* |
| PREV   NEXT | **FRAMES**    **NO FRAMES**     **All Classes** |


---
