# Supplementary material for: Semantic integration of gene expression analysis tools and data sources using software connectors
Source: BMC Genomics. 2013 Oct 25;14(Suppl 6):S2. doi: 10.1186/1471-2164-14-S6-S2 (PMC3908368; doi:10.1186/1471-2164-14-S6-S2)
Supplement: Additional File 3 — GELC API. GELC API binary code (jar format) and documentation (javadoc format). [file 1471-2164-14-S6-S2-S3.zip › documentation/index-files/index-3.html]

E-Index (GELC API)


---


|  |  |  |  |  |  |  |  |  |  |
| --- | --- | --- | --- | --- | --- | --- | --- | --- | --- |
| |  |  |  |  |  |  |  | | --- | --- | --- | --- | --- | --- | --- | | **Package** | Class | Use | **Tree** | **Deprecated** | **Index** | **Help** | | | *Gene Expression Library Class API v1.0* |
| **PREV LETTER**   **NEXT LETTER** | **FRAMES**    **NO FRAMES**     **All Classes** |


A C E G M R S T V 

---


## **E**

**equals(AbsoluteCDNAReadsCountingBasedValue)** - Method in class gelc.AbsoluteCDNAReadsCountingBasedValue: Compares this object against the specified object. **equals(AbsoluteIntensityBasedValue)** - Method in class gelc.AbsoluteIntensityBasedValue: Compares this object against the specified object. **equals(AbsoluteSAGETagsCountingBasedValue)** - Method in class gelc.AbsoluteSAGETagsCountingBasedValue: Compares this object against the specified object. **equals(CDNARead)** - Method in class gelc.CDNARead: Compares this object against the specified object. **equals(ExperimentalCondition)** - Method in class gelc.ExperimentalCondition: Compares this object against the specified object. **equals(Gene)** - Method in class gelc.Gene: Compares this object against the specified object. **equals(MatureTranscript)** - Method in class gelc.MatureTranscript: Compares this object against the specified object. **equals(RatioIntensityBasedValue)** - Method in class gelc.RatioIntensityBasedValue: Compares this object against the specified object. **equals(RelativeCDNAReadsCountingBasedValue)** - Method in class gelc.RelativeCDNAReadsCountingBasedValue: Compares this object against the specified object. **equals(RelativeSAGETagsCountingBasedValue)** - Method in class gelc.RelativeSAGETagsCountingBasedValue: Compares this object against the specified object. **equals(SAGETag)** - Method in class gelc.SAGETag: Compares this object against the specified object. **ExperimentalCondition** - Class in gelc: This class represents an experimental condition. **ExperimentalCondition(String)** - Constructor for class gelc.ExperimentalCondition: Constructor ExperimentalCondition.

---


|  |  |  |  |  |  |  |  |  |  |
| --- | --- | --- | --- | --- | --- | --- | --- | --- | --- |
| |  |  |  |  |  |  |  | | --- | --- | --- | --- | --- | --- | --- | | **Package** | Class | Use | **Tree** | **Deprecated** | **Index** | **Help** | | | *Gene Expression Library Class API v1.0* |
| **PREV LETTER**   **NEXT LETTER** | **FRAMES**    **NO FRAMES**     **All Classes** |


A C E G M R S T V 

---
