# Supplementary material for: Semantic integration of gene expression analysis tools and data sources using software connectors
Source: BMC Genomics. 2013 Oct 25;14(Suppl 6):S2. doi: 10.1186/1471-2164-14-S6-S2 (PMC3908368; doi:10.1186/1471-2164-14-S6-S2)
Supplement: Additional File 3 — GELC API. GELC API binary code (jar format) and documentation (javadoc format). [file 1471-2164-14-S6-S2-S3.zip › documentation/index-files/index-4.html]

G-Index (GELC API)


---


|  |  |  |  |  |  |  |  |  |  |
| --- | --- | --- | --- | --- | --- | --- | --- | --- | --- |
| |  |  |  |  |  |  |  | | --- | --- | --- | --- | --- | --- | --- | | **Package** | Class | Use | **Tree** | **Deprecated** | **Index** | **Help** | | | *Gene Expression Library Class API v1.0* |
| **PREV LETTER**   **NEXT LETTER** | **FRAMES**    **NO FRAMES**     **All Classes** |


A C E G M R S T V 

---


## **G**

**gelc** - package gelc: **Gene** - Class in gelc: This class represents a gene. **Gene(String)** - Constructor for class gelc.Gene: Constructor Gene. **GeneRegulation** - Enum in gelc: This class represents a specific representation of a ratio intensity-based gene expression value. **getValue()** - Method in class gelc.AbsoluteCDNAReadsCountingBasedValue: Obtains the absolute cDNA reads counting-based value itself. **getValue()** - Method in class gelc.AbsoluteIntensityBasedValue: Obtains the absolute intensity-based value itself. **getValue()** - Method in class gelc.AbsoluteSAGETagsCountingBasedValue: Obtains the absolute SAGE tags counting-based value itself. **getValue()** - Method in class gelc.CDNARead: Obtains the cDNA read value. **getValue()** - Method in class gelc.ExperimentalCondition: Obtains the experimental condition value. **getValue()** - Method in class gelc.Gene: Obtains the gene value. **getValue()** - Method in class gelc.MatureTranscript: Obtains the mature transcript value. **getValue()** - Method in class gelc.RatioIntensityBasedValue: Obtains the ratio intensity-based value itself. **getValue()** - Method in class gelc.RelativeCDNAReadsCountingBasedValue: Obtains the relative cDNA reads counting-based value itself. **getValue()** - Method in class gelc.RelativeSAGETagsCountingBasedValue: Obtains the relative SAGE tags counting-based gene expression value itself. **getValue()** - Method in class gelc.SAGETag: Obtains the SAGETag value.

---


|  |  |  |  |  |  |  |  |  |  |
| --- | --- | --- | --- | --- | --- | --- | --- | --- | --- |
| |  |  |  |  |  |  |  | | --- | --- | --- | --- | --- | --- | --- | | **Package** | Class | Use | **Tree** | **Deprecated** | **Index** | **Help** | | | *Gene Expression Library Class API v1.0* |
| **PREV LETTER**   **NEXT LETTER** | **FRAMES**    **NO FRAMES**     **All Classes** |


A C E G M R S T V 

---
