# Supplementary material for: Semantic integration of gene expression analysis tools and data sources using software connectors
Source: BMC Genomics. 2013 Oct 25;14(Suppl 6):S2. doi: 10.1186/1471-2164-14-S6-S2 (PMC3908368; doi:10.1186/1471-2164-14-S6-S2)
Supplement: Additional File 3 — GELC API. GELC API binary code (jar format) and documentation (javadoc format). [file 1471-2164-14-S6-S2-S3.zip › documentation/index-files/index-8.html]

T-Index (GELC API)


---


|  |  |  |  |  |  |  |  |  |  |
| --- | --- | --- | --- | --- | --- | --- | --- | --- | --- |
| |  |  |  |  |  |  |  | | --- | --- | --- | --- | --- | --- | --- | | **Package** | Class | Use | **Tree** | **Deprecated** | **Index** | **Help** | | | *Gene Expression Library Class API v1.0* |
| **PREV LETTER**   **NEXT LETTER** | **FRAMES**    **NO FRAMES**     **All Classes** |


A C E G M R S T V 

---


## **T**

**toString()** - Method in class gelc.AbsoluteCDNAReadsCountingBasedValue: Returns a string representation of this AbsoluteCDNAReadsCountingBasedValue object. **toString()** - Method in class gelc.AbsoluteIntensityBasedValue: Returns a string representation of this AbsoluteIntensityBasedValue object. **toString()** - Method in class gelc.AbsoluteSAGETagsCountingBasedValue: Returns a string representation of this AbsoluteSAGETagsCountingBasedValue object. **toString()** - Method in class gelc.CDNARead: Returns a string representation of this CDNARead object. **toString()** - Method in class gelc.ExperimentalCondition: Returns a string representation of this ExperimentalCondition object. **toString()** - Method in class gelc.Gene: Returns a string representation of this Gene object. **toString()** - Method in class gelc.MatureTranscript: Returns a string representation of this MatureTranscript object. **toString()** - Method in class gelc.RatioIntensityBasedValue: Returns a string representation of this RatioIntensityBasedValue object. **toString()** - Method in class gelc.RelativeCDNAReadsCountingBasedValue: Returns a string representation of this RelativeCDNAReadsCountingBasedValue object. **toString()** - Method in class gelc.RelativeSAGETagsCountingBasedValue: Returns a string representation of this RelativeSAGETagsCountingBasedValue object. **toString()** - Method in class gelc.SAGETag: Returns a string representation of this SAGETag object.

---


|  |  |  |  |  |  |  |  |  |  |
| --- | --- | --- | --- | --- | --- | --- | --- | --- | --- |
| |  |  |  |  |  |  |  | | --- | --- | --- | --- | --- | --- | --- | | **Package** | Class | Use | **Tree** | **Deprecated** | **Index** | **Help** | | | *Gene Expression Library Class API v1.0* |
| **PREV LETTER**   **NEXT LETTER** | **FRAMES**    **NO FRAMES**     **All Classes** |


A C E G M R S T V 

---
