# Supplementary material for: Semantic integration of gene expression analysis tools and data sources using software connectors
Source: BMC Genomics. 2013 Oct 25;14(Suppl 6):S2. doi: 10.1186/1471-2164-14-S6-S2 (PMC3908368; doi:10.1186/1471-2164-14-S6-S2)
Supplement: Additional File 3 — GELC API. GELC API binary code (jar format) and documentation (javadoc format). [file 1471-2164-14-S6-S2-S3.zip › documentation/overview-tree.html]

Class Hierarchy (GELC API)


---


|  |  |  |  |  |  |  |  |  |  |
| --- | --- | --- | --- | --- | --- | --- | --- | --- | --- |
| |  |  |  |  |  |  |  | | --- | --- | --- | --- | --- | --- | --- | | **Package** | Class | Use | **Tree** | **Deprecated** | **Index** | **Help** | | | *Gene Expression Library Class API v1.0* |
| PREV   NEXT | **FRAMES**    **NO FRAMES**     **All Classes** |


---


## Hierarchy For All Packages

**Package Hierarchies:**: gelc

---

## Class Hierarchy

- java.lang.Object
  - gelc.**AbsoluteCDNAReadsCountingBasedValue**- gelc.**AbsoluteIntensityBasedValue**- gelc.**AbsoluteSAGETagsCountingBasedValue**- gelc.**CDNARead**<E>- gelc.**ExperimentalCondition**- gelc.**Gene**- gelc.**MatureTranscript**- gelc.**RatioIntensityBasedValue**<E>- gelc.**RelativeCDNAReadsCountingBasedValue**<E>- gelc.**RelativeSAGETagsCountingBasedValue**<E>- gelc.**SAGETag**

## Enum Hierarchy

- java.lang.Object
  - java.lang.Enum<E> (implements java.lang.Comparable<T>, java.io.Serializable)
    - gelc.**GeneRegulation**

---


|  |  |  |  |  |  |  |  |  |  |
| --- | --- | --- | --- | --- | --- | --- | --- | --- | --- |
| |  |  |  |  |  |  |  | | --- | --- | --- | --- | --- | --- | --- | | **Package** | Class | Use | **Tree** | **Deprecated** | **Index** | **Help** | | | *Gene Expression Library Class API v1.0* |
| PREV   NEXT | **FRAMES**    **NO FRAMES**     **All Classes** |


---
